# Avian opioid peptides: evolutionary considerations, functional roles and a challenge to address critical questions

**DOI:** 10.3389/fphys.2023.1164031

**Published:** 2023-06-06

**Authors:** Krystyna Pierzchała-Koziec, Colin G. Scanes

**Affiliations:** ^1^ Department of Animal Physiology and Endocrinology, University of Agriculture in Krakow, Kraków, Poland; ^2^ Colin G. Scanes, Department of Biological Science, University of Wisconsin Milwaukee, Milwaukee, WI, United States

**Keywords:** enkephalin, dynorphin, nociceptin, birds, chicken

## Abstract

The present review considers the putative hormonal opioid peptides in birds. In birds and all other vertebrates, there are four opioid related genes encoding a series of peptides. These genes are, respectively, proenkephalin (PENK), prodynorphin (PDYN), pronociceptin (PNOC) and proopiomelanocortin (POMC). Proenkephalin (PENK) encodes Met- and Leu-enkephalin together with peptides containing met enkephalin motifs in birds, mammals and reptiles. Proopiomelanocortin (POMC) encodes β endorphin together with adrenocorticotropic hormone (ACTH), and melanocyte stimulating hormone (MSH). Prodynorphin (PDYN) encoding dynorphins A and B with α- and β-neoendorphins together intermediate polypeptides across the vertebrates. Pronociceptin (PNOC) encodes nociceptin together with possibly putative avian nocistatin and a non-opioid peptide derived from the C terminal of pronociceptin. There is a high degree of identity in the sequences of enkephalin peptides, dynorphin-A and B and nociceptin in birds and, to a less extent, across vertebrates. The opioid peptides exert effects related to pain together with other biological actions such as growth/development acting via a series of opioid receptors. What is unclear, particularly in birds, is the biological roles and interactions (additivity, antagonistic and synergistic) for the individual opioid peptides, the processing of the prohormones in different tissues and the physiological relevance of the different peptides and, particularly, of the circulating forms.

## 1 Introduction

This review will consider the neuropeptides from PENK, PDYN, and PNOC focusing on these in birds. With one exception, these peptides contain an enkephalin motif; this being an tetrapeptide with tyrosine–glycine—glycine–phenylalanine residues (YGGF). Moreover, a series of questions will be asked. It is noted that we have previous reviewed the peptides from POMC and, hence, these will not be discussed in the present discussion (Scanes and Pierzchała -Koziec, 2018; Scanes and Pierzchała -Koziec, 2021).

Four genes have been identified that encode opioid peptides. These are the following:

• Proenkephalin (PENK) encoding Met- and Leu-enkephalin,
• Proopiomelanocortin (POMC) encoding β-endorphin, adrenocorticotropic hormone (ACTH), and melanocyte stimulating hormone (MSH),
• Prodynorphin (PDYN) encoding Dynorphin-A and B together with α-Neoendorphin and β-Neoendorphin,
• Pronociceptin (PNOC) encoding Nociceptin/Orphanin FQ together with a putative avian nocistatin and a possibly biologically active C terminal peptide (reviewed: Bu et al., 2020; Dhaliwal and Gupta, 2022).

There is some evidence for other opioid like peptides. For example, Zadina and colleagues (1997) reported two peptides with opioid activity, namely, endomorphin-1 (YPWF-NH_2_) and endomorphin-2 (YPFF-NH_2_). However, a gene(s) encoding endomorphin-1 and endomorphin-2 has not been yet identified. Other endomorphin-like peptides have been reported, specifically mexneurin 1 (Mx 1), Mx 2, and Mx 3. These are encoded by prepromexneurin (Matus-Ortega et al., 2017).

There are four major types of opioid receptors. These G protein-coupled receptors include the following:

• Delta opioid receptors (DOR) binding Met- and Leu-enkephalin,
  o Sub-types
    ⁃ Delta 1
    ⁃ Delta 2
• Mu opioid receptors (MOR) binding β endorphin together with both endomorphin 1 and 2,
  o Sub-types
    ⁃ Mu 1
    ⁃ Mu 2
    ⁃ Mu 3
• Kappa opioid receptors (KOR) binding dynorphin-A and B,
  o Sub-types
    ⁃ Kappa 1
    ⁃ Kappa 2
    ⁃ Kappa 3
• Nociceptin receptors (NOR) (naloxone insensitive) binding nociceptin

(reviewed: Bu et al., 2020; Dhaliwal and Gupta, 2022). In addition, there is a zeta opioid receptor (reviewed Dhaliwal and Gupta, 2022).

### 1.1 Evolution of PENK, PDYN, PNOC, and POMC genes

Proenkephalin, prodynorphin, pronociceptin, and proopiomelanocortin (POMC) not only share enkephalin motifs but also cysteine residues at similar points in their sequences (reviewed: Fricker et al., 2022). The basis for four opioid genes and, also, the four receptors are two separate gene duplication early in vertebrate evolution (Sundström et al., 2010).

### 1.2 Converting enzymes

Proenkephalin, prodynorphin, and pronociceptin can be cleaved by a series of cysteine proteases/thiol proteases/convertases acting at both monobasic and dibasic sites. This generates a series of neuropeptides depending on the presence and specificity of the convertases. Different tissues can have different expression levels of proenkephalin and/or prodynorphin and/or pronociceptin and different convertases generating a series of neuropeptides (Day et al., 1998).

## 2 Proenkephalin (PENK) and the derived enkephalin neuropeptides

### 2.1 Structures and processing of proenkephalin and enkephalin neuropeptides derived from proenkephalin

Met-enkephalin is a pentapeptide with the following sequence of amino-acid residues: tyrosine–glycine-glycine- phenylalanine–methionine (YGGFM). Similarly, Leu-enkephalin is a pentapeptide with the following sequence of amino-acid residues: tyrosine–glycine-glycine- phenylalanine–leucine (YGGFL).

**FIGURE 1:**
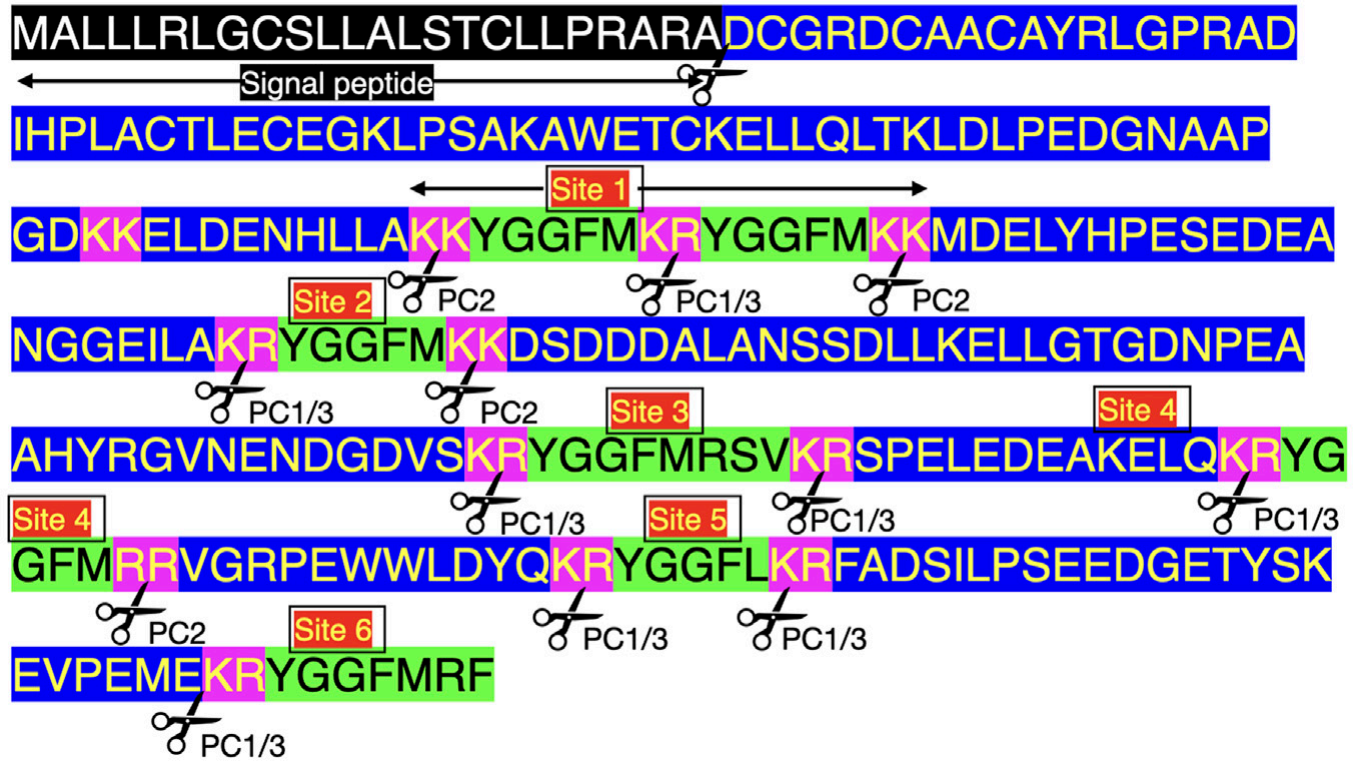
Structure of chicken preproenkephalin (deduced from mRNA Genbank XM_040664746). Key: PC prohormone convertase Pink highlighted indicates pairs of basic amino acid residues Green highlighted indicates enkephalin motifs Black highlighted indicates signal peptide Blue highlight other amino acid residues.

There are seven enkephalin motifs (YGGFM/L with two basic amino-acid residues adjacent to the ends of the motif) in avian proenkephalin (see Figure 1; Supplementary Figure S1). Proenkephalin can processed into the following:

• YGGFM x 4 [sites 1, 2, and 4 (Figure 1)]
• YGGFL [Site 5 (Figure 1)]
• YGGFMRF or YGGFMR [Site 6 (Figure 1)].

The possibility of additional biologically active products of proenkephalin are discussed below under “Biological activity of Met-enkephalin and other proenkephalin derived peptides”.

There are also both glycosylation and phosphorylation sites within proenkephalin (reviewed: Fricker et al., 2022).

### 2.2 Evolution of enkephalin peptides

Four enkephalin peptides (YGGFM x 4, YGGFL, and YGGFMRF/YGGFMR), have identical sequences in birds, mammals and reptiles. Moreover, there are the same flanking basic amino acid pairs (Table 1). In contrast, the peptide (YGGFMRSI or YGGFMRSV) in birds and reptiles differs from that in mammals (Supplementary Figure S1). The regions of proenkephalin that are not part of enkephalin peptides show little variation across the class Aves and in reptiles (see Supplementary Figure S1).

**TABLE 1:** Enkephalin peptides with flanking pairs of basic amino acid residues.

	Site 1	Site 2	Site 3	Site 4	Site 5	Site 6
Enkephalin						
Aves	YGGFM x 2	YGGFM	YGGFMRSI	YGGFM	YGGFL	YGGFMRF
Mammalia			YGGFMRGL or YGGFMKSA			
Reptilia			YGGFMRSI			
Amphibia			YGGFMRDY or YGGFMRGS		YGGFM	
Non-tetrapod Sarcopterygii			YGGFMRSL			
Actinopterygii	YGGFM & YGGFT or YGGFMI	YGGFI or absent	e.g., YGGFM		Absent	YGGFMGY or YGGFMD
Chondrichthyes	YGGFM x 2	YGGFM	YGGFMNGF		YGGFM	YGGFMRI
Pairs of flanking basic amino acid residues						
Aves	KK & KK with KR between 2 motifs	KR & KK	KR & KR	KR & RR	KR & KR	KR
Mammalia						
Reptilia						
Amphibia					KR & RR	
Non-tetrapod Sarcopterygii	KR & KK with KR between 2 motifs					
Actinopterygii	KK & KK with KR between 2 motifs				Not applicable	
Chondrichthyes		KR & KK			KR & KR	

It is noted that there are degenerate enkephalin motifs and/or the absence of flanking pairs of basic amino acid residues in both boney and cartilaginous fish (see Table 2; Supplementary Figure S1). Leu-enkephalin is not present in non-tetrapod sarcopterygians, actinopterygian fish or cartilaginous (Chondrichthyes) fish with Met-enkephalin replacing it (Table 2).

**TABLE 2:** Comparison of enkephalin motifs in proenkephalin and prodynorphin in vertebrate classes.

	Enkephalin motifs^a^	Met-enkephalin Motifs^b^	Leu-enkephalin Motifs^c^	Degenerate motifs^d^
Proenkephalin				
Mammalia	7	6	1	0
Aves	7	6	1	0
Reptilia	7	6	1	0
Amphibia	7	6	1	0
Non-tetrapod sarcopterygians	7	7	0	0
Actinopterygii	5	5	0	2
Chondrichthyes	7	7	0	0
Prodynorphin				
Mammalia	3	0	3	0
Aves	4	2	1	1
Reptilia	5	3^5^	1	0
Amphibia	4	2	2	0
Non-tetrapod sarcopterygians	4	0^5^	3	0
Actinopterygii	5	1^6^	2–3	1–2
Chondrichthyes	5	1–3	1	1–2

^a^ YGGFM/L.

^b^ K/RK/RYGGFMK/RK/R.

^c^ K/RK/RYGGFLK/RK/R.

^d^ GGFM, or YGGF, or GGF, with or without flanking pairs of basic amino-acid residues.

^e^ Plus YGGFF.

^f^ Plus YGGFI.

### 2.3 Converting enzymes and proenkephalin

There are multiple converting enzymes generating neuropeptides from proenkephalin (see Figure 1). For instance, cathepsin L in secretory vesicles in chromaffin granules converts proenkephalin to enkephalins (Yasothornsrikul et al., 2003).

### 2.4 Biological activity of Met-enkephalin and other proenkephalin derived peptides

It is reasonable to assume that Met- and Leu-enkephalin play a role in reducing pain and associated responses in mammals (Cullen and Cascella, 2022; Dhaliwal and Gupta, 2022) and birds (Scanes and Pierzchała-Koziec, 2018).

There are also negative effects of Met-enkephalin on growth and development. For instance, Met-enkephalin inhibits angiogenesis in the chorioallantoic membrane of chick embryo with the effect reduced in the presence of naltrexone (Blebea et al., 2000). Moreover, Met-enkephalin exerts an anti-proliferative effect on cultured adrenocortical cells (rat: Malendowicz et al., 2005). Furthermore, Met-enkephalin depresses proliferation of peripheral blood T cells based in the elevated proliferation following application of anti-sense oligonucleotide (humans: Kamphuis et al., 1998). In contrast, Met-enkephalin stimulated proliferation by human peripheral lymphocytes (Hucklebridge et al., 1989).

**FIGURE 3:**
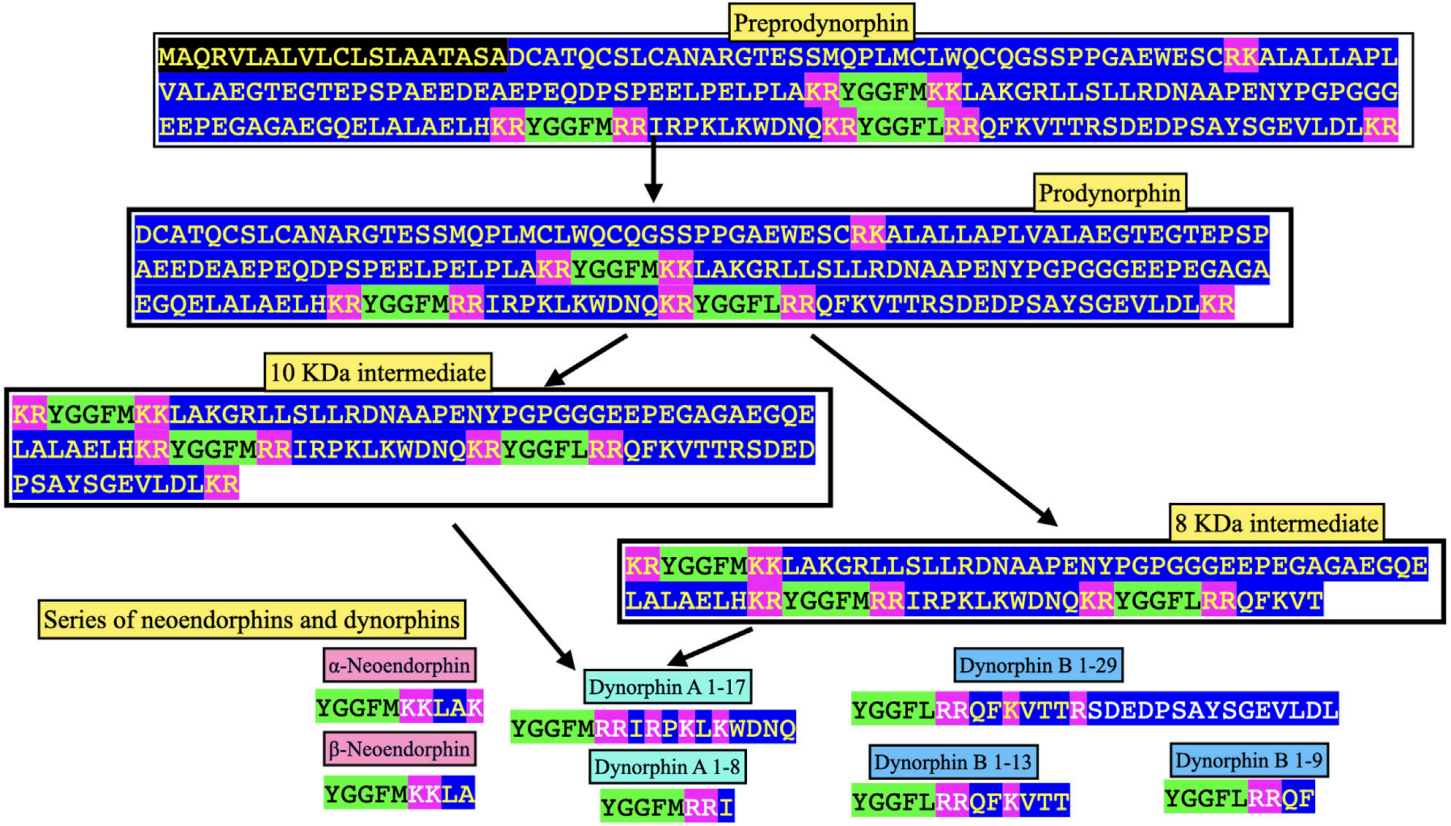
Processing of chicken prodynorphin. Key: Green highlight indicates enkephalin motif Pink highlight indicates amino-acid residue pairs Black highlight indicates signal peptide Blue highlight other amino acid residues.

It is assumed that proenkephalin derived peptides act via δ opioid receptors; with met- and leu-enkephalin having similar activities in both a cortical acetylcholine release assay in rats (Jhamandas and Sutak, 1980) and evoking a response with chicken δ opioid receptors (Bu et al., 2020). While this is probably the case with Met-and Leu-enkephalin, there is evidence that other proenkephalin derived peptides act via κ or μ opioid receptors. Fragments of proenkephalin have been isolated from bovine adrenal medullary tissue; these containing at least one enkephalin motif (Mizuno et al., 1980a; Mizuno et al., 1980b; Kilpatrick et al., 1981) (Table 3). Superficially, it would be thought that these endogenous fragments that are smaller and closer to the enkephalin would have greater biological activity. However, that is not the case. In fact, the longer the fragments, the greater their activities are in a guinea pig ileum assay (Table 3) (Kilpatrick et al., 1981). These might be dismissed as irrelevant to a discussion of avian opioids. What suggests that these peptides are important to avian physiology is that the sequences of these putative fragments in chickens as an exemplar bird, the chicken, are identical to those in cattle (Table 3; Figure 3; Supplementary Figure S1). Were these not to be functional, random mutations would have been expected in 315 million years since the last common ancestor of birds and mammals (Irisarri et al., 2017). Without selective pressure, these would be incorporated to proenkephalin.

**TABLE 3:** Biological activity of a series of cattle opioid peptides in the guinea pig ileum assay (data calculated from Kilpatrick et al., 1981).

Opioid peptides	Mammalian peptide	Potency in guinea pig ileum assay^a^
Met-enkephalin	YGGFM	1.0
Leu-enkephalin	YGGFL	0.071
β-endorphin	YGGFMTSEKSQTPLVTLFKNAIIKNAHKKGQ^b^	1.16
Dynorphin A 1–13	YGGFLRRIRPKLK	69.2
Extended enkephalin containing peptides (mammalian)		
BAM12^c^	YGGFMRRVGRPE	2.3
BAM20^c^	YGGFMRRVGRPEWWMDYEKR	16.4
BAM22^c^	YGGFMRRVGRPEWWMDYEKRYG	27.7
Peptide E	YGGFMRRVGRPEWWMDYEKRYGGFL	37.5

^a^ Acting via κ opioid receptors.

^b^ GeneBank accession XM_019970607.

^c^ Mizuno et al., 1980a; Mizuno et al., 1980b

### 2.5 Circulating and tissue concentrations of Met-enkephalin

Table 4 summarizes plasma and tissue concentrations of Met-enkephalin in chickens. Plasma concentrations of native Met-enkephalin (free, five amino acids peptide) were similar in male and female chickens (Table 4). Plasma concentrations of cryptic Met-enkephalin (total, Met-enkephalin released from proenkephalin by enzymatic hydrolysis) were 15.7 fold higher than those of native Met-enkephalin (Table 4).

**TABLE 4:** Plasma and tissue concentrations of Met-enkephalin in 14 weeks old chickens (based on data in Pierzchala- Koziec and Mazurkiewicz-Karasińska, 2016).

Tissue	Mean ± SEM	
	Female	Male
Plasma (pmoles L^−1^)		
Native Met-enkephalin	50 ± 7.9	50 ± 8.4
Cryptic Met-enkephalin	813 ± 98	758 ± 61
Tissue native Met enkephalin concentrations (pmoles g^−1^)		
Anterior pituitary gland	797 ± 119^d^	986 ± 128^c^
Hypothalamus	329 ± 49^c^	101 ± 11^b^**
Adrenal gland	135 ± 15^b^	144 ± 20.0^b^
Heart atria	3.6 ± 0.1^a^	5.2 ± 1.1^a^
Heart ventricles	2.4 ± 0.6^a^	3.6 ± 0.1^a^
Kidney	1.7 ± 0.2^a^	2.9 ± 0.6^a^

^a,b,c,d^ Different superscript letter indicates difference *p < 0.05 between tissues by one way ANOVA, and Tukey’s test.

Sex difference **p < 0.01.

What are the possibilities? It may be a proenkephalin, or fragments of proenkephalin (as in Table 1) or intermediate forms in the processing of proenkephalin as is seen with dynorphin (see section below—Converting Enzymes and Prodynorphin).

The highest concentrations of Met-enkephalin in chickens are in the hypothalamus, adrenal gland and anterior pituitary gland (see Table 4) (Pierzchała-Koziec and Mazurkiewicz-Karasińska, 2016).

### 2.6 Stress and circulating concentrations of Met-enkephalin

In mammals, there is strong evidence that plasma concentration of immunoreactive Met-enkephalin are elevated by stresses such as insulin induced hypoglycemia in sheep (Owens et al., 1988), induction of diabetes in rats (Kolta et al., 1992), acute induction of hypotension in anesthetized dogs (Mason et al., 1987) and restraint stressed rats (Barron et al., 1990).

Similarly, both plasma concentrations of native Met-enkephalin and adrenal expression of PENK (proopiomelanocortin) were elevated in female chickens subjected to restraint. Water deprivation did not affect either native or cryptic Met-enkephalin but depressed adrenal concentrations of Met-enkephalin. Plasma concentrations of native but not cryptic Met-enkephalin were increased in feed deprived immature female chickens. Morphine challenge was followed by depressed plasma and adrenal concentrations of both native and cryptic Met-enkephalin together with decrease adrenal expression of PENK in female chickens (Pierzchała-Koziec and Mazurkiewicz-Karasińska, 2016). Plasma concentrations of native Met-enkephalin were increased in young chickens stressed by crowding while adrenal concentrations of Met-enkephalin were depressed by crowding.

It is generally assumed that plasma is the compartment of blood in which hormones are found. However, in mammals, Met enkephalin is reported to be produced by leukocytes (human: Kraemer et al., 2013). Similarly, Met-enkephalin is reported to be synthesized by peripheral blood T cells and monocytes (humans: Kamphuis et al., 1998). Production of Met-enkephalin by monocytes was increased in the presence of lipopolysaccharide (humans: Kamphuis et al., 1998). An additional possibility is that fragments of proenkephalin and/or Met- or Leu-enkephalin are generated at the target tissue level.

### 2.7 Differential processing and release of proenkephalin derived neuropeptides

There is strong evidence that basal and stressed induced circulating concentrations of proenkephalin derived neuropeptides can be and, often are, different. The late fetal increases in circulating concentrations of Met-enkephalin were smaller than those for Met-enkephalin-arginine-phenylalanine (MERF) (Simonetta et al., 1993). Moreover, induction of hypotension in fetal sheep was accompanied by markedly greater increase in circulating concentrations of MERF than those of Met-enkephalin (Mateo et al., 1995). Similarly, asphyxia is followed by increases in circulating concentrations of MERF in fetal sheep (Coulter et al., 1990). Furthermore, there were increases circulating concentrations of MERF but not Met-enkephalin in hypoxic fetal sheep (Simonetta et al., 1996). There is not information on the differential release of different enkephalin peptides in birds.

### 2.8 Questions

What is not known are the following:

1. The extent to which different enkephalin peptides are released and whether this varies with different tissues.
2. The biological activities of different proenkephalin peptides to avian opioid receptors and exert agonist or even antagonist effects on different derived neuropeptides in avian tissues. In particular, are any of the peptides derived from proenkephalin capable of activating avian μ opioid receptors.
2. Whether cryptic Met-enkephalin represents intermediates in the proteolytic cleavage and/or proenkephalin.
3. Whether circulating concentration of enkephalin reflects hormonal mode of action or do they reflect “spill over” from neural, paracrine or autocrine effects?
4. The physiological control of the release of enkephalin and other products of cleavage of proenkephalin in birds.
5. Whether avian leukocytes or, for that matter, erythrocytes and thrombocytes, produce Met-enkephalin or other products of cleavage of proenkephalin and contribute to plasma concentrations of enkephalin neuropeptides.

## 3 Prodynorphin (PDYN) and the derived neuropeptides

### 3.1 Structure of neuropeptides derived from prodynorphin

**FIGURE 2:**
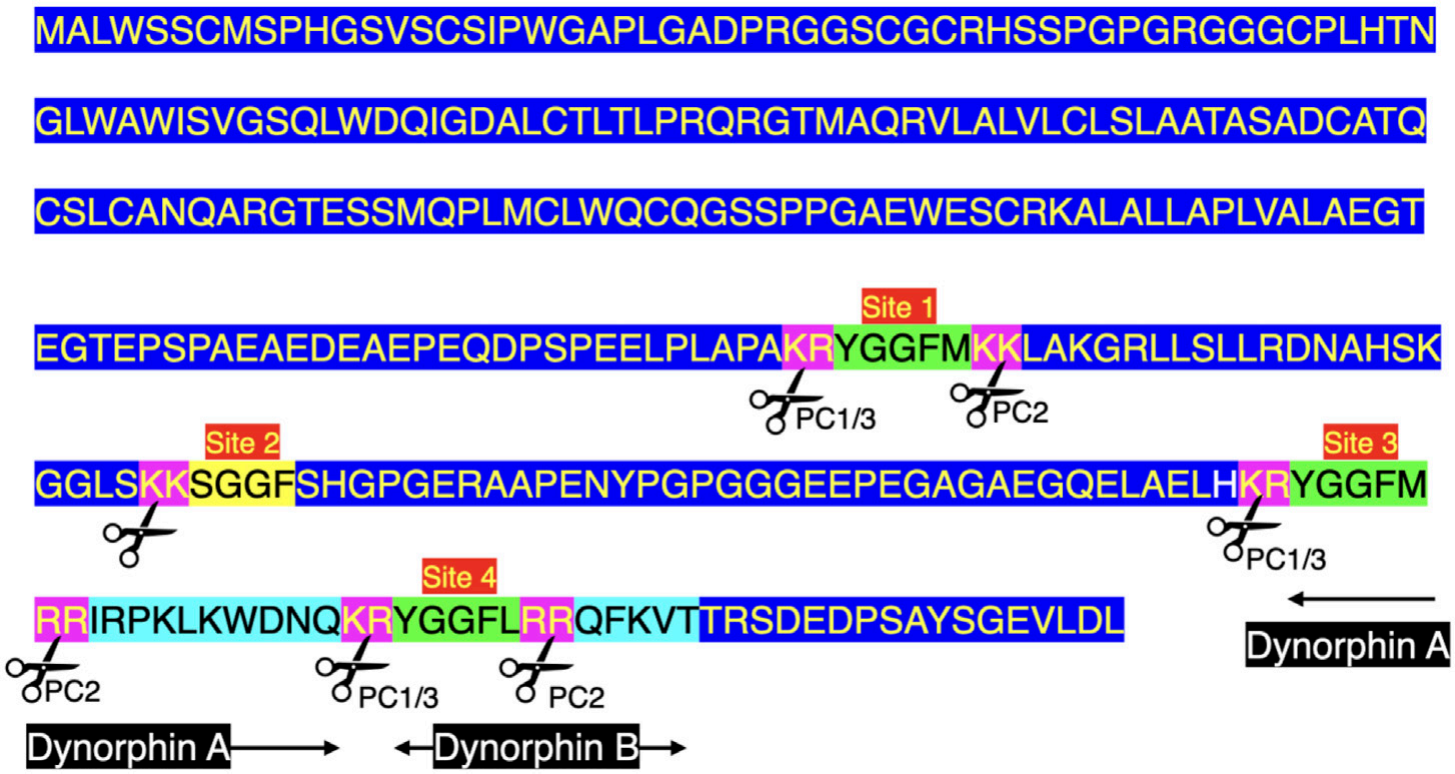
Structure of chicken preprodynorphin (deduced from mRNA Genbank XM_040650978). Key: PC prohormone convertase Green highlight indicates enkephalin motif Light blue highlight indicates peptide sequences found in neuropeptides along with enkephalin motif Pink highlight indicates flanking basic amino-acid residues pairs Yellow highlight indicates degenerate enkephalin motif e.g., lacking two basic amino-acid residue pairs on either N or C sides of the enkephalin motif or lacking enkephalin motif Blue highlight other amino acid residues.

There are three enkephalin motifs together with one putative or degenerate enkephalin motif in prodynorphin in chickens (Figure 2) and other birds (Supplementary Figure S2). Similarly, there are four enkephalin motifs/degenerate enkephalin motifs in birds (Supplementary Figure S2). There are five enkephalin motifs in prodynorphin in reptiles and the lungfish (Supplementary Figure S2). Moreover, there are five enkephalin motifs/degenerate enkephalin motifs in boney fish (Supplementary Figure S2). In contrast, there are only three enkephalin motifs in mammals (Supplementary Figure S2). It is suggested that in five enkephalin motifs represents the ancestral form with motifs and/or the flanking basic amino acid residue pairs lost during tetrapod evolution. In contrast, there are seven enkephalin motifs in proenkephalin (Figure 1; Supplementary Figure S1).

**FIGURE 4:**
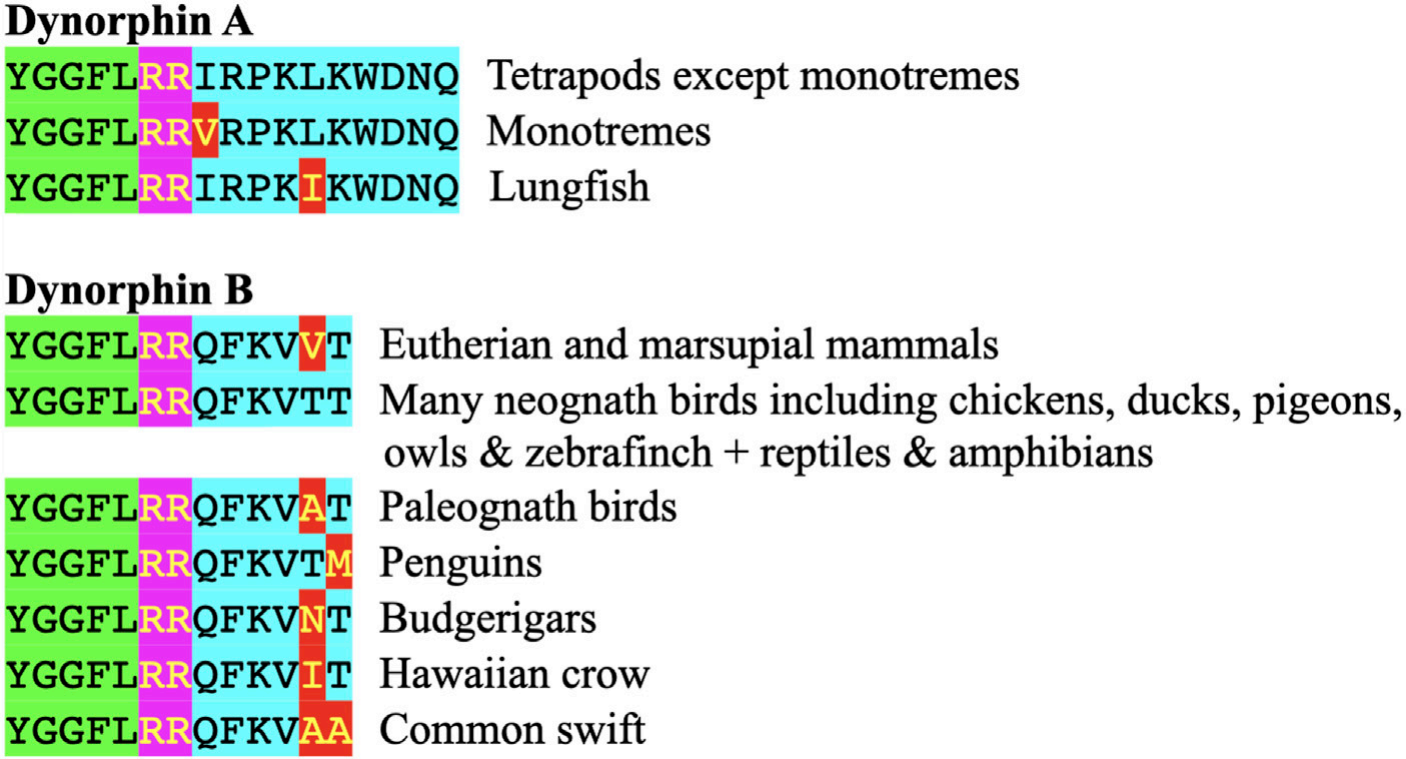
Comparison of the structures of dynorphin A and dynorphin B in vertebrates. Key: Green highlight indicates enkephalin motif Pink highlights indicate basic amino acid pair (putative site for proteolysis) Red highlight indicates different amino acid residue from that in chickens, many other birds, reptiles and amphibians; this being the presumptive ancestral form Light blue highlight indicates additional amino acid residues in dynorphin A or B.

Figure 3 summarizes the processing of prodynorphin. In mammals, there are three enkephalin motifs with flanking basic amino acid residues. These are processed into opioid neuropeptides: α- and β - neoendorphins and dynorphins A and B (Figures 3, 4; Supplementary Figure S2).

In chickens, other avian species, and cold-blooded vertebrates, there are sequences of amino acid residues with flanking pairs of basic amino-acid residues that could generate both dynorphin A and dynorphin B (see Figures 4; Supplementary Figure S2).

Despite the degree of identity between dynorphin A and B in their homologues across tetrapods, the regions of prodynorphin that are not part of dynorphin neuropeptides exhibit considerable variation even across the class Aves (Figure 4; Supplementary Figure S2).

### 3.2 Evolution of dynorphin

The sequences of the neuropeptides dynorphin A and dynorphin B, are remarkably conservative across vertebrate classes (see Figure 3; Supplementary Figure S2). The enkephalin motifs in proenkephalin and dynorphin across the vertebrates are compared in Table 2. In contrast, there are marked differences in the sequences between avian α- (or β-) neoendorphin and mammalian or reptilian α- (or β-) neoendorphin (Figure 4).

Dynorphin A has an identical structure in eutherian and placental mammals, reptiles, birds and amphibians (see Figure 3; Supplementary Figure S2). There are single substitutions in monotremes and lungfish (see Figure 3; Supplementary Figure S2).

Dynorphin B, there is an identical structure in some avian species including chickens, ducks and pigeons together with reptiles and amphibians (see Figures 3, 4; Supplementary Figure S2). This suggested that this is the ancestral form of dynorphin B in tetrapods. Compared to the structure of dynorphin B in many neognath birds, there are only single amino-acid residue substitutions (but not the same one) with those in eutherian and placental mammals and also in birds of the Infra-class *Paleognathae*, (see Figure 4; Supplementary Figure S2). There are three differences of the 13 amino-acid residues between lungfish (YGGFLRRHFKITV) compared to those of tetrapods.

Monotremes do not appear to have dynorphin B with the reported sequence exhibiting marked degeneracy compared to dynorphin B in other tetrapods.

• YGASRPRPFKPVT Platypus,
• YGAVRPRPYKLVA Australian echidna.

Moreover, in at least one amphibian species (*Microcaecilia unicolor*), dynorphin B may not be present based on the absence of dibasic cleavage site in prodynorphin (see Supplementary Figure S2).

Despite the degree of identity between dynorphin A and B in their homologues across tetrapods, the regions of prodynorphin that are not part of dynorphin neuropeptides exhibit considerable variation even across the class Aves (see Supplementary Figure S2).

### 3.3 Converting Enzymes and Prodynorphin

Prodynorphin is subject to proteolysis by convertase(s) generating neoendorphins and dynorphins together with potentially Leu-enkephalin in mammals (Berman et al., 1999; reviewed Ner and Silberring, 2013) and both Met- and Leu-enkephalins in birds. In mammals, there is evidence that prodynorphin is cleaved in a disparate manner in different regions of the brain and pituitary gland (Cone et al., 1983; Seizinger et al., 1994a; Seizinger et al., 1994b).

In mammals, the principal cleavage products of prodynorphin are the following:

• α-Neo-endorphin (YGGFLRKYPK).
• β-Neo-endorphin (YGGFLRKYP).
• Dynorphin A 1–17 (YGGFLRRIRPKLKWDNQ).
• Dynorphin A 1–8 (YGGFLRRIR.
• Dynorphin B 1–29 (YGGFLRRQFKVVTRSQEDPSAYYEELFDV)

(e.g., Seizinger et al., 1984b). Other putative neuropeptides include dynorphin B 1–13 (YGGFLRRQFKVVT), dynorphin B 1–9 (YGGFLRRQF), 8 and 10 KDa prodynorphin intermediates (see Figure 4) and, potentially, Leu-enkephalin (Day et al., 1998). The predicted structures of avian neuropeptides cleavage products of avian prodynorphin are shown in Figure 3. There are differences between avian and mammalian prodynorphin, comparing prodynorphin products in chickens and cattle. For instance, there are Met-enkephalin motifs in both the putative avian α-/β-neoendophin and dynorphin A neuropeptides instead of Leu-enkephalin motifs in mammals.

### 3.4 Biological activity of dynorphin

Dynorphin B had a potency of 700 compared to Leu-enkephalin in a guinea pig ileum longitudinal muscle assay; the effects of dynorphin B being blocked by naloxone (Goldstein et al., 1979. The chicken κ opioid receptor is activated by dynorphin A and B (Bu et al., 2020).

### 3.5 Release of dynorphin

There is limited information on the release of dynorphin in mammals and none in birds. There is release of both immunoreactive dynorphin and α-neoendorphin from the perfused rat duodenum *in vitro* (Corbett et al., 1988). Release of α-neoendorphin and dynorphin were increased in the presence of nicotine in cultured human phaeochromocytoma cells (Yanase et al., 1987); this action presumably acting via nicotinic cholinergic receptors.

### 3.6 Circulating concentrations of dynorphin

There are limited reports on plasma concentrations of dynorphin or neoendorphin in mammals (humans: Shen and Wang, 1998; Moniaga et al., 2019; Shahkarami et al., 2019) with basal concentrations of 13.1 pmol L^−1^ (Moniaga et al., 2019). The plasma concentrations of dynorphin A were increased in pilots subjected to hypoxia (Shen and Wang, 1998). To the best of our knowledge, there are no reports of plasma concentrations of dynorphin in either poultry or wild birds. Plasma concentrations of IR-dynorphin have been reported in human volunteers as 40.3 ± 6.4 pmol L^−1^ (calculated from Margioris et al., 1990) but markedly lower in control subjects being compared to heart transplant patients [3.3 ± 0.2 pmol L^−1^] (calculated from Ationu et al., 1993). Plasma concentrations of IR-dynorphin were elevated in human subjects receiving administration of hypertonic saline (Margioris et al., 1990).

Another compartment of blood, leukocytes, have been demonstrated to synthesize dynorphin in mammals. For instance, preprodynorphin expression is reported in peripheral blood cells (Shahkarami et al., 2019). There are no reports of leukocytic expression of preprodynorphin in leukocytes or for that matter in erythrocytes or thrombocytes in birds.

### 3.7 Stress and circulating concentrations of prodynorphin derived neuropeptides in birds

Plasma concentrations of an immune-reactive α-neoendorphin have been reported in an abstract:

• Adult female chickens 11.5 ± 0.86 pmol L^−1^.
• Adult male chickens 15.9 ± 0.57 pmol L^−1^ (calculated from Pierzchala and Przewlocki, 1989).

Plasma concentrations of α-neoendorphin in pullets were increased following crowding stress. This effect is blocked by the prior administration of naltrexone (Pierzchała-Koziec et al., 1996).

### 3.8 Neoendorphin and dynorphin: expression, tissue concentrations and release

There is no information on the expression of the prodynorphin together with tissue distribution and release outside of the brain or on circulating concentrations of either dynorphin A and B in birds. There is some information on these in mammals. High concentrations of IR-dynorphin are detected in the mammalian posterior pituitary gland (reviewed: Margioris et al., 1990) with marked expression in magnocellular neurons in the hypothalamus that project into the posterior pituitary gland (Sherman et al., 1986a). Moreover, expression of pro-dynorphin shifts in a manner similar to that of vasopressin in supraoptic and paraventricular nuclei (Sherman et al., 1986b). Also, there is increased expression of the prodynorphin gene in the hypothalamus of dehydrated or salt loaded rats (Sherman et al., 1986a).

Both dynorphin and α-neoendorphin are released from rat duodenal tissue *in vitro* (Majeed et al., 1987). Moreover, release of IR-dynorphin and IR α-neoendorphin is increased by serotonin (rat: Majeed et al., 1987). There is no information on the distribution in tissues or the control of the release of dynorphin A or B in any avian species.

### 3.9 Questions

What is not known are the following:

1. The forms of dynorphin A and B together with neoendorphin that are produced by various avian tissues.
2. The biological activities of different prodynorphin derived peptides in birds.
3. Are circulating concentration of prodynorphin derived neuropeptides exerting hormonal effects or do they reflect “spill over” from neural, paracrine or autocrine effects?
4. The physiological control of dynorphin A and B together with neoendorphin release.
5. Whether avian leukocytes or, for that matter, erythrocytes and thrombocytes, produce prodynorphin derived neuropeptides and contribute to their concentrations in the plasma.
6. The extent to which different neuropeptides are released and whether this varies with different tissues.

## 4 Pronociceptin (PNOC) and the derived neuropeptide nociceptin (orphanin FQ)

### 4.1 Introduction to nociceptin

**FIGURE 5:**
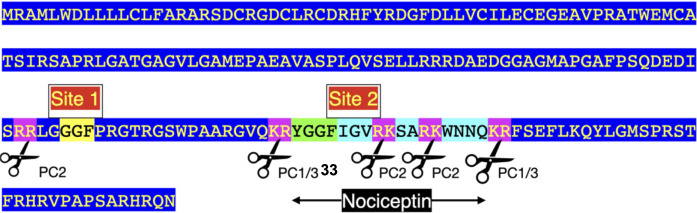
Structure of chicken prepronociceptin (Genbank XM_040697232). Key: PC prohormone convertase Green highlight indicates enkephalin motif Pink highlights indicate basic amino acid pair (putative site for proteolysis) Yellow highlight indicates degenerate enkephalin motif e.g., lacking two basic amino-acid residue pairs on C flanking of the partial enkephalin motif. Light blue highlight indicates additional amino acid residues in nociceptin Blue highlight other amino acid residues.

The structure of pronociceptin is shown in Figure 5. There is peptide containing an enkephalin motif generated from the prepronociceptin mRNA in mammals, birds and reptiles. In contrast, there are two peptides with enkephalin motifs encoded by prepronociceptin mRNA in boney and cartilaginous fish, amphibians and lungfish. Nociceptin is the ligand for the nociceptin opioid receptor; this being insensitive to naloxone.

### 4.2 Structure and evolution of nociceptin

**FIGURE 6:**
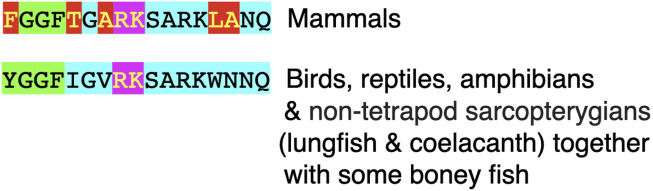
Structure of nociceptin in vertebrates. Key Green highlight indicates enkephalin motif (YGGF) or partial motif with flanking pairs of basic amino acids residues both N and C terminals. Pink highlight indicates a pair of basic amino acid residues. Red highlight indicates differences with the sequence of amino acid residues relative to that in birds, reptiles, amphibians and non-tetrapod sarcopterygians Light blue highlights indicate other amino-acid residues.

Nociceptin is a neuropeptide with 17 amino-acids (see Figure 4; Supplementary Figures S3, S4). There is an identical structure for nociceptin across mammalian species (see Figure 6; Supplementary Figures S3, S4). This differs from other tetrapods with five substitutions of amino acid residues (see Figure 4). The last common ancestor for mammals and reptiles/birds is estimated as living 315 million years ago (during the Carboniferous period) (Irisarri et al., 2017). In contrast, there are identical structures for nociceptin in non-mammalian tetrapods together with non-tetrapod Sarcopterygii (lungfish and coelacanths) (see Figure 6; Supplementary Figures S3, S4). The last common ancestor for tetrapods and non-tetrapod Sarcopterygii is estimated as living 428 million years ago (in the Silurian period) (Irisarri et al., 2017).

What is particularly surprising is the substitution of tyrosine to phenylalanine residues in mammalian nociceptin (Figure 4; Supplementary Figure S3). This is an unique case of such a substitution and is not found with any other neuropeptides that include enkephalin motifs.

### 4.3 Nomenclature

Nociceptin/orphanin FQ (N/OFQ) is a reasonable name in mammals due to the presence of phenylalanine (F) at the N terminal and a glutamine (Q) at the C terminal. However, in other tetrapods together with lungfish, this is not appropriate. Instead, it is more accurate to refer the avian neuropeptide as nociceptin/orphanin YQ [due to the N terminal being tyrosine (Y) and glutamine being the C terminal] or simply as nociceptin.

### 4.4 Biological role of nociceptin

Both the chicken nociceptin and κ opioid receptors are activated by nociceptin (Bu et al., 2020). It might be assumed that nociceptin is exerting an analgesic effect in birds. In addition, nociceptin plays a role in embryonic development. Ectodermal expression of nociceptin is increased by somatostatin with nociceptin playing a role in the formation of placode progenitors in chick embryos (Lleras-Forero et al., 2013). However, there are no studies on the effects of avian nociceptin on either avian physiology or pathology. There are, however, reports of the effect of mammalian nocicentin in birds (see below). If nociceptin was not important, it is difficult to envision why an identical structure is found across the tetrapods together with non-tetrapod Sarcopterygians. Thus, the structure was retained in its entirety through at least 428 million years since the last common ancestor of tetrapods and non-tetrapod Sarcopterygians (Irisarri et al., 2017). In addition, an identical structure is found in some boney fish (Figure 6; Supplementary Figures S3, S4).

### 4.5 Nociceptin and the hypothalamic control of feeding in birds

In young meat line chickens, intracerebroventricular injection of mammalian nociceptin was followed by increased consumption of feed (Abbasnejad et al., 2005; Zendehdel et al., 2013; Zendehdel et al., 2015; Zendehdel et al., 2017; Zendehdel et al., 2019). There is evidence for beta-adrenergic, serotoninergic, dopaminergic and histaminergic involvement in feeding in chickens induced by mammalian nociceptin. Nociceptin induced feed intake was increased after prior administration of a β_2_ adrenergic antagonist (Zendehdel et al., 2017). However, there was no evidence for α- or β_1_ or β_3_ adrenergic involvement in mammalian nociceptin induced feed consumption (Zendehdel et al., 2017). Nociceptin induced feed consumption was increased by either pharmacological blocking serotonin (5-HT) synthesis or a 5-HT receptor 2 antagonist (Zendehdel et al., 2013). In addition, prior administration of the dopamine precursor, L-DOPA depressed mammalian nociceptin induced feeding while a D_2_ dopamine antagonist increase the response to N/OFQ (Zendehdel et al., 2019). Moreover, the effect of mammalian nociceptin was increased by prior administration of an H_1_ histamine antagonist but not a H_2_ antagonist (Zendehdel et al., 2015). In contrast, the effect of mammalian nociceptin on feed consumption was decreased by prior administration of an H_3_ histamine antagonist (Zendehdel et al., 2015). It is cautioned that mammalian nociceptin differs by five amino-acid residues out of a total of 17 compared to that in other tetrapods (Figure 6). Studies employing avian nociceptin are needed. If nociceptin was not important, it is difficult to envision why an identical structure is found across the tetrapods except for mammals (Figure 6; Supplementary Figures S3, S4).

### 4.6 Circulating concentrations of nociceptin

There are reports of plasma concentrations of nociceptin in humans but not in other mammals or birds. Post operative plasma concentrations of nociceptin have been reported as 39 pmol L^−1^ in individuals with intravenous patient-controlled analgesia (Lee and Jeon, 2013). In contrast, plasma concentrations of nociceptin were 0.55 pmol L^−1^ in patients with sepsis and 1.7 pmol L^−1^ in patients with sepsis who subsequently died (Williams et al., 2008). There is a need for examination of plasma concentrations of nociceptin in birds under both a series of physiological and pathological situations.

### 4.7 Nociceptin tissue concentrations and release

There is no information on the distribution in tissues or the control of the release of nociceptin in any avian species.

### 4.8 A putative avian nocistatin

In mammals, pronociceptin also encodes a second biologically active peptide, nocistatin (Okuda-Ashitaka et al., 1998; reviewed; Fricker et al., 2022). This is generated by proteolytic cleavage at basic amino-acid residue pairs (Okuda-Ashitaka et al., 1998; reviewed; Fricker et al., 2022) (see Figure 5). The length of nocistatin exhibiting marked variability (bovine: 17; human: 31 amino-acid residues). The C–terminal for nocistatin in humans and cattle consists of the following hexapeptide: EQKQLQ (Meunier et al., 1995; Nothacker et al., 1996; Okuda-Ashitaka and Ito, 2000). Based on a PubMed search, there are no reports of nocistatin in birds. However, there are predicted sequences of a putative avian nocistatin in a series of birds (see Figures 5, 6 also see Supplementary Figure S5). These are separated by basic amino-acid residue pairs. In birds, these are characterized by having an identical or very similar C terminal hexapeptide, AARGVQ; this being found in, for instance, in Okarito brown kiwi (XM_026067016), chicken (XM_040697232) and Hawaiian crow (XM_048298403) together with similar hexapeptides as AAKGVQ (common canary—XM_050971908) and TARGVQ (California condor—XM_050895080).

### 4.9 A putative avian non-opioid neuropeptide derived from pronociceptin

Fricker et al. (2022) included non-opioid peptide(s) derived from pronociceptin when nocicentin was cleaved (Figures 5, 6). This viewed these likely to be biologically active (Fricker et al., 2022). This was presumably a neuropeptide as are all the peptides derived from proenkephalin, prodynorphin, and pronociceptin (Fricker et al., 2022).

It was questioned whether such a putative biologically active peptide might exist in birds and, also, in reptiles. The deduced sequence for nociceptin has a pair of basic amino-acid residues (lysine-arginine) at both its N and C terminal in birds and reptiles (Figure 5; Supplementary Figure S3) together with mammals (Fricker et al., 2022). Proteolytic cleavage would be expected to occur at these sites generating nociceptin and a second peptide(s) again in birds, reptiles and mammals (Fricker et al., 2022).

There is strong similarity between the N terminal of the putative peptide with identical residues at positions 1, 2, 3, 4, 7, 8, and 9 in mammals (e.g., human and Tasmanian devil) compared to birds (e.g., chicken and kiwi) (Supplementary Figure S6). This would have 30 or 31 amino-acid residues (also see Supplementary Figure S6). This putative C terminal neuropeptide would be considered as non-opioid as it lacks the YGGF motif (Figure 6).

Comparison of the putative peptide from deduced structures of pronociceptin in birds suggested that there are two structures of the nociceptin C terminal non-opioid peptide in birds:

• FSEFLKQYLGMSPRSTFRHRVPAPSARHRQN in chickens (with the V replaced by an I in some species).
• FSEFLKQYLGMSPRSSEYDIAGGISEHNEI (Supplementary Figure S6).

They share a 15 amino-acid residue peptide (FSEFLKQYLGMSPRS). The C terminal of each in different species have multiple cases of identical amino-acid residues (Supplementary Figure S6).

What was unexpected that both forms were found in avian species in the Infra-order Paleognathae and Neognathae, in the Clade Neoaves, in the Clade Australaves (e.g., members of the orders Passeriformes, and Falconiformes), in the Clade Afroaves (Strigiformes and Accipitriformes) and within both the orders Piciformes and Passeriformes (avian classification and evolution based on Brusatte et al., 2015). This is not consistent with a simplistic evolutionary interpretation. The explanation for this is not readily apparent.

### 4.10 Questions

What is not known includes the following:

1. Which forms of nociceptin and other putative neuropeptides derived from pronociceptin are produced by various avian tissues.
2. What are the biological activities of peptides derived from pronociceptin are produced by various avian tissues.
3. Are circulating concentration of nociceptin exerting hormonal effects or do they reflect “spill over” from neural, paracrine or autocrine roles?
4. The physiological control of the release of nociceptin and other peptides derived from pronociceptin.
5. Whether avian leukocytes or, for that matter, erythrocytes and thrombocytes, produce nociceptin and other peptides derived from pronociceptin contribute to plasma concentrations of nociceptin.

## 5 Conclusion

What is almost completely missing in avian species is information on cleavage pattern of proenkephalin, prodynophin and pronociceptin in different tissues and the relative activities of the multiple endogenous opioids/peptides via the δ-opioid, κ-opioid and μ-opioid receptors in chickens or other birds. Moreover, given the multiplicity of opioid peptides and their roles, it is questioned whether at least some are released in response to the welfare challenges such as stress or injury. It is assumed that the biologically active peptides derived from proenkephalin are Met-enkephalin and Leu-enkephalin. However, other peptides are derived from proenkephalin and, based on mammalian studies, they have markedly different biological activities. The sequence of amino-acid residues in these peptides is identical in birds and mammals arguing for their importance. Avian prodynorphin is likely to be subject to proteolytic cleavage generating dynorphin A and B and, probably also an avian neoendorphin with the first two exhibiting marked similarity in sequence. Avian pronociceptin is cleaved to produce nociceptin, nocistatin and a non-opioid C terminal peptide with the first and third having close homology with their mammalian counterparts. (Pierzchala and Van Loon, 1990; Chen et al., 2007; Thompson et al., 2014; Avenali et al., 2017).
