# Supplementary material for: Avian opioid peptides: evolutionary considerations, functional roles and a challenge to address critical questions
Source: Front Physiol. 2023 Jun 6;14:1164031. doi: 10.3389/fphys.2023.1164031 (PMC10280075; doi:10.3389/fphys.2023.1164031)
Supplement: Supplementary file 1 [file DataSheet4.DOCX]

**Mammals**

FGGFTGARKSARKLANQ Human (*Homo sapiens*) (XM_005273532)

FGGFTGARKSARKLANQ European shrew (*Sorex araneus*) (XM_004614742)

FGGFTGARKSARKLANQ Tasmanian devil (*Sarcophilus harrisii*) (XM_012540067)

FGGFTGARKSARKLANQ Platypus (*Ornithorhynchus anatinus*) (XM_029053275)

**Reptiles**

YGGFIGVRKSARKWNNQ Green anole (*Anolis carolinensis*) (XM_008121138)

YGGFIGVRKSARKWNNQ Burmese python (*Python bivittatus*) (XM_007425961)

YGGFIGVRKSARKWNNQ Western painted turtle (*Chrysemys picta*) (XM_005308457)

YGGFIGVRKSARKWNNQ Chinese alligator (*Alligator sinensis*) (XM_006029253)

YGGFIGVRKSARKWNNQ American alligator (*A. mississippiensis*) (XM_019483807)

**Birds**

YGGFIGVRKSARKWNNQ Emu (*Dromaius novaehollandiae)* (XM_026102004)

YGGFIGVRKSARKWNNQ Okarito brown kiwi (*Apteryx rowi*) (XM_026067016)

YGGFIGVRKSARKWNNQ Chicken (*Gallus gallus)* (XM_040697232)

YGGFIGVRKSARKWNNQ Mallard (*Anas platyrhynchos*) (XM_027455447)

YGGFIGVRKSARKWNNQ Emperor penguin (*Aptenodytes forsteri)* (XM_009283460)

YGGFIGVRKSARKWNNQ Condor (*Gymnogyps californianus*) (XM_050895080)

YGGFIGVRKSARKWNNQ Hawaiian crow (*Corvus hawaiiensis*) (XM_048298403)

YGGFIGVRKSARKWNNQ Zebra finch (*Taeniopygia guttata)* (XM_041714961)

**Amphibians**

YGGFIGVRKSARKWNNQ Common toad (*Bufo bufo*) (XM_040430952)

YGGFIGVRKSARKWNNQ *Microcaecilia unicolor* (XM_030195556)

**Non-tetrapod Sarcopterygii**

YGGFIGVRKSARKWNNQ Lungfish (*Protopterus annectens*) (XM_044061856)

YGGFIGVRKSARKWNNQ Coelacanth (*Latimeria chalumnae*) (XM_006003069)

**Some from Class Actinopterygii e.g.**

YGGFIGVRKSARKWNNQ Silver crucian carp (*Carassius gibelio*) (XM_052580621)

Some from **Class Chondrichthyes e.g.**

YGGFMGVRKSIRNWSNLSRQTNQ Smaller spotted catshark (Scyliorhinus canicula)

(XM_038800484)

**Supplementary Figure 4.** Structure of nociceptin in vertebrate species together with non-tetrapod Sarcopterygians.

Green highlight indicates enkephalin motif (YGGF) or partial motif. Pink highlight indicates a dibasic site. Red highlight indicates differences with the sequence of amino acid residues relative to that in neognath birds.
