# Supplementary material for: Avian opioid peptides: evolutionary considerations, functional roles and a challenge to address critical questions
Source: Front Physiol. 2023 Jun 6;14:1164031. doi: 10.3389/fphys.2023.1164031 (PMC10280075; doi:10.3389/fphys.2023.1164031)
Supplement: Supplementary file 2 [file DataSheet6.DOCX]

FSEFLKQYLGMSPRSTFRHRVPAPATRHRQN Kiwi

FSEFLKQYLGMSPRSTFRHRVPAPATRHRQN Swan

FSEFLKQYLGMSPRSTFRHRVPAPSARHRQN Chicken

FSEFLKQYLGMSPRSTFRHRIPAPSARHRQN Kittiwake

FSEFLKQYLGMSPRSTFRHRIPAPSARHRQN Woodpecker

FSEFLKQYLGMSPRSTFRHRIPAPSARHRQN Swift

FSEFLKQYLGMSPRSTFRHRIPAPSARHRQN Kestrel

FSEFLKQYLGMSPRSTFRHRIPAPSARHRQ Condor

FSEFLKQYLGMAPRSTFRHRFPAPSARHRQN Crow

FSEFLKQYLGMSPRSTFRHRIPAPSARHRQN Owl

FSEFLKQYLGMSPRSTFRHRIPAPSARHRQN Manakin

FSEFLKQYLGMAPRSTFRHRLPA---RPRPN.Red-winged blackbird

FSEFLKQYLGMAPRSTFRHRLPA---RHRQN Village indigobird

FSEFLKQYLGMSPRSSEYDIA-ADLNEHNEI Emu

FSEFLKQYLGMSPRSSEYEVAGGVS-EHNEI Rock pigeon

FSEFLKQYLGMSPRSSEYDIAGGIS-EHNEI Honeyguide

FSEFLKQYLGMSPRSSEYDIAGGIS-EHNEI Common cuckoo

FSEFLKQYLGMSPRSSEYDIAGGIS-EHNE Penguin

FSEFLKQYLGMSPRSSEYDIAGGIS-EHNEI Goshawk

FSEFLKQYLGMSPRSSEYDIAGGIS-EHNEI Eagle

FSEFLKQYLGMSPRSSEYDIAGGIS-EHNEI Whooping crane

FSEFLKQYLGMAPRSSEYGR-GG-AGDANEI Cowbird

FSEFLKQYLGMAPRSSEYGR-GGGAGDTNEI Canary

FSEFLKQYLGMSPRSSEYDGIGNDLNEQNEI Chinese alligator

FSEFLKQYLGMSPRSSEYDGIGNDLNEQNEI American alligator

**SUPPLEMENTARY FIGURE 6.** Comparison of the deduced sequence of amino-acid residues of the C terminal peptide generated from pronociceptin in birds and crocodylia. Blue highlighting indicates deduced sequence in chicken. Red highlighting indicates different from chicken.

Infraclass: Paleognathae: Kiwi - Okarito brown kiwi (*Apteryx rowi*) (XM_026067016);

Infraclass: Neognathae:

Superorder Galloanserae:

Chicken (*Gallus gallus)* (XM_040697232) (order: Galliformes); Swan - Black swan (*Cygnus atratus*) (XM_035563714) (order: Anseriformes).

Clade: Neoaves:

Kittiwake - Black-legged kittiwake (*Rissa tridactyla*) (XM_054196450) (order: Charadriiformes); Woodpecker (*Dryobates pubescens*) (XM_054179755) (order: Piciformes); Swift - common swift (*Apus apus*) (XM_051614450) (order: Apodiformes)

Kestrel - Lesser kestrel (*Falco naumanni*) (XM_040599078) (order: Falconiformes), Condor - California condor (*Gymnogyps californianus*) (XM_050895080) (order: Cathartiformes);

Crow - Hawaiian crow (*Corvus hawaiiensis*) (XM_048298403) (order: Passeriformes);

Barn owl (*Tyto alba*) (XM_042803621) (order: Strigiformes), Manakin - White-collared manakin (*Manacus candei*) (XM_051809817) (order: Passeriformes); and Village indigobird (*Vidua chalybeata*) (XM_053932981) (order: Passeriformes).

Infraclass: Paleognathae: Emu (*Dromaius novaehollandiae)* (XM_026102004);

Infraclass: Neognathae:

Clade: Neoaves:

Rock pigeon (*Columba livia*) (XM_021298518) (order: Columbiformes); Honeyguide - Greater honeyguide (*Indicator indicator)* (XM_054383818) (order: Piciformes); Common cuckoo (*Cuculus canorus*) (XM_009563767) (order: Cuculiformes); Penguin - Emperor penguin (*Aptenodytes forsteri)* (XM_009283460) (order: Sphenisciformes); Goshawk: orthern goshawk (*Accipiter gentilis*) (XM_049819329) (order: Accipitriformes); Eagle - Harpy eagle (*Harpia harpyja*) (XM_052810340) (order: Accipitriformes); Whooping crane (*Grus americana)* XM_054821560) (order: Gruiformes);  Brown-headed cowbird (*Molothrus ater*) (XM_036381213) (order: Passeriformes); Common canary (*Serinus canaria*) (XM_050971908) (order: Passeriformes); Chinese alligator (*Alligator sinensis*) (XM_006029253) and American alligator (*Alligator mississippiensis*) (XM_019483807).
