# Supplementary material for: Avian opioid peptides: evolutionary considerations, functional roles and a challenge to address critical questions
Source: Front Physiol. 2023 Jun 6;14:1164031. doi: 10.3389/fphys.2023.1164031 (PMC10280075; doi:10.3389/fphys.2023.1164031)
Supplement: Supplementary file 3 [file DataSheet3.DOCX]

MKVLLCDLLLLSLFSSVFSSCQRDCLTCQEKLHPALDSFDLEVCILECEEKVFPSPLWTPCTKVMARSSWQLSPAAPEHVAAALYQPRASEMQHLRRMPRVRSLFQEQEEPEPGMEEAGEMEQKQLQKRFGGFTGARKSARKLANQKRFSEFMRQYLVLSMQSSQRRRTLHQNGVQVIPQTACAQPQTCRLGIRIPSSPRH

Human (*Homo sapiens*) (XM_005273532 )

MKRLLCGLLLLSLCPGRTSGSCHRACLSCREKLGPALDSFSLEVCVLQCEGQGLSGPLWTPCTEATAGDSWQLDPTGSEQVGGAPETQQLKRMPRVRSLGQEQERPEPGPQDVQQKRLQKRFGGFTGARKSARKLANQKRFSEFMRQYLVLSMQSSQRRRTWRQKAH

European shrew (*Sorex araneus*) (XM_004614742)

MRTLFYDLLLLGLFSNVFSDCQKDCLTCREKLHPSRDSFNVDACISDCEGRDLLGAFWGPCTKAVNRGPGPLGPASKAKAAAASYQPRGMEAAEAPASHGPAEAKNRVSRIRSLFRGPEPEAAESLGEAGEEMQKKLQKRFGGFTGARKSARKLANQKRFSEFMRQYLVMSMHASERQRQGLAPGRALRQNLSV

Tasmanian devil (*Sarcophilus harrisii*) (XM_012540067)

MKTVVRGLVLLGLVSCALSHCQRECSWCRARLGPAVTTFSTTACILECQGLVPAQASWGTCARIPRSAPGRARPARRSEEEVDEEEDEEKEEEEEEEEEEEEGATPWTAPWTLTPVEARPGPGPGPGPGPGPGSGPGPWGLGPTPGRGVQKRFGGFTGARKSARKLANQKRVSEFVRQFVMLSLQSSERHNRLPAALRARAHL

Platypus (*Ornithorhynchus anatinus*) (XM_029053275)

MKTVVRGLVLLGLVSCALSHCQRECSWCKARLGPAVTTFSTTACILECQGLVPAQASWATCARIPRSAPGRKRFGGFTGARKSARKLANQKRVSEFVRQFVMLSLQSSERHNRLPAALRARAH

Australian echidna (*Tachyglossus aculeatus*) (XM_038771448)

MRAMLWDLLLLCLFARARSDCRGDCLRCDRHFYRDGFDLLVCILECEGEAVPRATWEMCATSIRSAPRLGATGAGVLGAMEPAEAVASPLQVSELLRRRDAEDGGAGMAPGAFPSQDEDISRRLGGGFPRGTRGSWPAARGVQKRYGGFIGVRKSARKWNNQKRFSEFLKQYLGMSPRSTFRHRVPAPSARHRQN

Chicken (*Gallus gallus)* (XM_040697232)

MRAVLWDLLLLLWLCARARGDCRGDCAHCERQLYRDRFDVLICILECEGQAVPRATWELCAAAAARAAPRPRRLSGRPWPSGGSPRRQEDEEEEEEEQEDISRRPGAGRAAKGVQKRYGGFIGVRKSARKWNNQKRFSEFLKQYLGMAPRSSEYGRGGGAGDTNEI

Common canary (*Serinus canaria*) (XM_050971908)

MRAVLWDLLLLCLFARVRGDCRGDCLRCDRNLYRDSFDVLVCILECEGEAVPRATWELCAAAAGRAAPRPRDLQDAGDPWHGAVAAVPAVPVSPLQVSELLRRREAEDEGVEPAPGAFPQPPEDISRRLDGFPRGTHGSRPAPTARGVQKRYGGFIGVRKSARKWNNQKRFSEFLKQYLGMSPRSTFRHRIPAPSARHRQ

California condor (*Gymnogyps californianus*) (XM_050895080)

MRAVLWDLLLLCLFAQARGDCRGDCLHCDRQLYRDSFDVLICILECEGEAVPRATWELCAAASRAAPRPRDLAAEPWPGAAPALPAGPANPLQVTELLRRRGADADEAAPGTFPQPPEEDISRRLGRFPPGMGGWRPAPAAKGVQKRYGGFIGVRKSARKWNNQKRFSEFLKQYLGMAPRSTFRHRFPAPSARHRQN

Hawaiian crow (*Corvus hawaiiensis*) (XM_048298403)

MRAVLWDLLLLCLFAHVLGDCQRDCLSCDRHAYSQWDGFNVLGHTYYTCLPTCTVHIWPHALCMPSPMCTARGVQKRYGGFIGVRKSARKWNNQKRFSEFLKQYLGMSPRSSEYDIAADLNEHNEI

Emu (*Dromaius novaehollandiae)* (XM_026102004)

MRAVLWDLLLLCLFAHVLGDCQRDCLSCDRHMYSQWDGFNVLVCVLECEGQAVPRATWELCAAASAGPREEEEEDDDDDDNDPADVVAQRPGARPPAEPRRLAAPAARGVQKRYGGFIGVRKSARKWNNQKRFSEFLKQYLGMSPRSTFRHRVPAPATRHRQN

Okarito brown kiwi (*Apteryx rowi*) (XM_026067016)

MRAVLWDLLLLCLFARARGDCRGDCLRCDRHLYRDSFDVLVCILECEGEAVPRATWELCAAAAGVQKRYGGFIGVRKSARKWNNQKRFSEFLKQYLGMSPRSSEYDIAGGISEHNE

Emperor penguin (*Aptenodytes forsteri)(*XM_009283460)

**Class Reptilia**

MRALLRDLLLLCLLAHVLGDCQRDCLACSRLVYNQPDSFNVLVCIVECEGKVFSSGTWELCSKVAGKATLQLSADSLEEDVYQPLDTEDGDLFGGGLKRYNDLTKVVDLSKVEDEKRVSKVSGLIRQREAEDGASDGSETQVGDFPEQPKDISKRLGDFLNGKYSYRQVLEPAVSGVQKRYGGFIGVRKSARKWNNQKRFSEFLKQYLGMSPRSSEYDGIGNDLNEQNEI

Chinese alligator (*Alligator sinensis*) (XM_006029253)

MRALLRDLLLLCLLAHVLGDCQRDCLACSRLVYNQPDSFNVLVCIVECEGKVFSSGTWELCSKVAGKATLQLSADSLEEDVYQPLDAEDGDLFGGGLKRYNDLTKVVDLSKVEDEKRVSKVSGLIRQREAEDGASDGSETQVGDFPEQPKDISKRLGDFLNGKYSYRQVLEPAVSGVQKRYGGFIGVRKSARKWNNQKRFSEFLKQYLGMSPRSSEYDGIGNDLNEQNEI

American alligator (*Alligator mississippiensis*) (XM_019483807)

MWILLRTSLLFLAFAYALSSDCRKDCLNCHRHLYSQQDDFSLLICVMECEKKLLSRATWDLCNKAIIRKPSSLLLGFEGLDDEVTWPFEFWDVGSPRGRGSLKRFGDFPRAAEDEKHVMRRPEPEDEPSHSSQADLLGDLLSDPGVQDLQKRFGGFIGVRKSARKWHNQKRFSEFLKQYLGMSPRSVEYDGMADGDLKEQNEV

Green anole (*Anolis carolinensis*) (XM_008121138)

MYTVYIYTHHCDRDTVEESHIRGSLATDNTPSGLATATMGALLWDLLLLCLFAHVLGDCQRDCLSCNRHLYNQQDNFNVLVCIMECEGKAFSSTTWELCTKVAGKSSIQVSADSLEEDSYQPLEMDDSGLFRGSRKHFDDLTKVVDLSKVEGEKRVSKVSSLIRQREVDEGVSNGSETPLGDFQEQPKDISKRLGGFLKGKYSYRQVLEPTVQGVQKRYGGFIGVRKSARKWNNQKRFSEFLKQYLGMSPRSSEYDSLTNDLNEQNEI

Western painted turtle (*Chrysemys picta*) (XM_005308457)

**Class Amphibia**

MKALLWSITLLSLLSHVLCDCQNDCVTCNKRLYQQDNFDTLVCIVECEGKIYSSSMWGVCKTVLIKSSIRLSMDSFEEDFKPFNVEDSQLTGDFKRLSDLTKVVDLSKIKDEKRLSKISNLIHEQGEEEDTSIDGGETALGMSAGTDELGDLQDPTNAMAKRFGGFVKGKYSYRKFMGPSKDLQKRYGGFIGVRKSARKWNNQKRFSEFLRQYLGMSTRSVDYDSFTNDMNELNEQNEI

Common toad (*Bufo bufo*) (XM_040430952)

MKTLLWTLVLLCLFANVLSDCQRDCLICKKHLYQQDDFNTLVCIVECEGEAYSSVIWKMCKKVLKKTAVQLSVDSMEDNTYNPLETEDGLFRSNLKHLEDLTKVVDLSKIENEKRISKISGLIQEQEEDNTIERSETPLHMTSEPDAFSDLQGQSRDITKRYGGFLKGKYSYRKFLEPARGMQKRYGGFIGVRKSARKWNNQKRFSEFLKQYLGMSTRSSGYDSLSADINEENEI

*Microcaecilia unicolor* (XM_030195556)

**Non-tetrapod Sarcopterygians**

MKTMFVTLLLLLCLFARVHGDCQKDCLACSKHLYQYSTFDALVCILECEVKVSSSEVWEACKRTLVVTVGTDSLGVDTYSPPEIMTPRNDGHLFESTMKHFGDITKVTDMDRKESEKRVAKAGLIRDREEEEEEDSFSNGNESLLEMDNDRNTIYGPSEPSHEISKKFGGFLKGKYSYRKLLEPGRGIQKRYGGFIGVRKSARKWNNQKRFSEFLKQYLGMSTRSSEYDSVSADINEQNEI

West African lungfish (*Protopterus annectens*) (XM_044061856)

**Class Actinopterygii**

MKTPLWTLLLLGLCNPAWCDCQKDCLFCSQKLHNEYAFNNLVCLVECHGKLSPGDTWEMCRRTTVEQNPKALLTVGYGVLKRAEEEADTSLPVDQDDGQGSETLQRFAHITQALGTDDQDQDMQLSKKYKFLQVQSAQESEEERDGDNETEGDEEEAAVHLIKRFGGFLKNKYGYRKFIDPGRSLQKRYGGFIGVRKSARKWNNQKRFSEFLKQYLGMSTRASEFNSMSADVTQQNE

Silver crucian carp (*Carassius gibelio*) (XM_052580621)

MKWSLWSLLLLCLCTPGRSDCQGDCLTCGLLLSNPQSQQQAFNTLVCLLECDGHVSPALTWDLCQRAMLPQYPLPTDDSAVSKRPEEKLSLYLAPADLESDGKMLYSAAMERYQQDGAEQEDEALVRRDAQYDSSPLGLTAEGDSLALGMENGEEEEKEEEVRRRRSGQGEGGDEEEDAVVQLTKRFGGFLKGRHSYRKLIGSPVRKSLQKRLGGFIGIRKSARKWNNQKRVSQLLRQYLGMTSSSTPGRGVGRFTNPAQGLRRLPSRL

Atlantic salmon (*Salmo salar*)( XM_014143699)

MKTPLWTLLLMGLFVHGRSDCQRDCLTCSQILPKDHGFDTLVCMVECHGAVYPGLTWERCQTALEEEPLASLSVGNPMPKRAEEEVETVLPMGQSDGALTYSGTLQRFDHVARALGLDELNQENQISQFSTAVQPQYEQEEHDAADWEIKSDQEGEPVNLTKRFGGFLKSKYGYRKFMDPGRSMQKRYGGFIGVRKSSRKWNNQKRFSEFLKQYLGMTTRASKFNSVSTDITRQNKV

Channel catfish (*Ictalurus punctatus*) (XM_017476286)

MKTPFWTLLLLCLCASSHSDCQGDCLTCGLILPEHQAFNTLVCILECEAQASPALTWDLCYQAAGLKHLPLPLQDEETSKRSDDEAEPVATVSIENDNGVEYTEALERFRHALQPSEELEKLTASYDPDLDPRTQEDQDDLGDEKSDDAAVSVSKRFGGFMKGRHGLRKLVSSGRPLQKRYGGFIGIRKSARKWNNQKRVSQLLRQYLSLTGRSGRSGHINSLSTRIRRQSE

Zebrafish (*Danio rerio*) (NM_001015044.1)

**Class Chondrichthyes**

MRTSLLSLILILYLVPCCQGDCAAECLTCNKLVYQHQHFNVLICILQCKGQVTSSVIWDNCRKSGSASSVLPLGVSTSQIGSHSFGSAGNNGRGNSGSTRGFLQIMKIMLPNGSMEGISDDGAEATFEVDNSQQDTKMPSDESSYGSRAEPLASNQYGGFMERKPNFREVAEGNRSFQKRYGGFMGVRKSIRNWSNLSRQTNQKRYSKFLRQYLGLATRSAEYDSLPGNLAV

Smaller spotted catshark (Scyliorhinus canicula) (XM_038800484)

MKTSLLSLAVLFHLIYSCQGDCKAECLTCKNLYQQEQFNILVCNLECEGKVPSSLMWKNCRNIISVSPLLPLKLSTSQARSLPFGVARIFGTLGNTHDVLQAVKNMSPIRSTEDIYDDEAEVNSSPQDTVMPSDKRSYDFQAESLASKPNERSMKRNHDISNAVESKKSFQKRYGGFMGIRKSARNWSNLIRQTSQKRYSKFLRQYLGLTTRSTDDSLPGNLAA

Smalltooth sawfish *(Pristis pectinate*) (XM_052024850)

**Supplementary Figure 3.** Comparison of the structure of prepronociceptin across vertebrates

Green highlight indicates either nociceptin peptide with enkephalin (YGGFM/YGGFL) or another peptide containing an enkephalin motif and both with flanking pairs of basic amino acid residues

Yellow highlight indicates degenerate enkephalin motifs.

Pink highlight indicates a dibasic site.

Light blue highlight indicates amino acid residues in peptide

Blue highlight indicates other amino acid residues.
