# Supplementary material for: Avian opioid peptides: evolutionary considerations, functional roles and a challenge to address critical questions
Source: Front Physiol. 2023 Jun 6;14:1164031. doi: 10.3389/fphys.2023.1164031 (PMC10280075; doi:10.3389/fphys.2023.1164031)
Supplement: Supplementary file 4 [file DataSheet5.DOCX]

PRVRSLFQEQEEPEPGMEEAGEMEQKQLQ Human

-----------LAAPAARGVQ Kiwi

-LGGGFPRGTRGSWPAARGVQ Chicken

LDGFPRGTHGSRPAPTARGVQ Condor

LGRFPPGMGGWRPAPAAKGVQ Hawaiian crow

----------PGAGRAAKGVQ Canary

SUPPLEMENTARY FIGURE 5. Comparison of the deduced sequences of nocistatin in examples of birds with that of humans.

Human – Avenali et asl., 2017; Kiwi – Okarito brown kiwi (XM_026067016), chicken (XM_040697232), Condor - California condor (XM_050895080), Crow - Hawaiian crow (XM_048298403), canary – common canary (XM_050971908).
