# Supplementary material for: Avian opioid peptides: evolutionary considerations, functional roles and a challenge to address critical questions
Source: Front Physiol. 2023 Jun 6;14:1164031. doi: 10.3389/fphys.2023.1164031 (PMC10280075; doi:10.3389/fphys.2023.1164031)
Supplement: Supplementary file 5 [file DataSheet2.DOCX]

MAWQGLVLAACLLMFPSTTADCLSRCSLCAVKTQDGPKPI

NPLICSLQCQAALLPSEEWERCQSFLSFFTPSTLGLNDKE

DLGSKSVGEGPYSELAKLSGSFLKELEKSKFLPSISTKEN

TLSKSLEEKLRGLSDGFREGAESELMRDAQLNDGAMETGT

LYLAEEDPKEQVKRYGGFLRKYPKRSSEVAGEGDGDSMGH

EDLHEDLYKRYGGFLRRIRPKLKWDNQKRYGGFLRRQFKV

VTRSQEDPNAYSGELFDA

Human (*Homo sapiens*) XM_011529244

MAWRGLVLAACLLLLPCATADCLSQCSSCAVKTQSGPKPV

NFLVCCLECQAALLPAKEWERCQNLQPLLTPFPLYPNGKE

DSETRAEPYGELATRPGPSLEDVEKIRIFLGPPKEESALG

RPRAGGAKSGLIGYMQPEDEFLEAAAAVASEENPKEQIKR

YGGFLRKYPKRSSEVAGEEDGEKEGHEDLYKRYGGFLRRI

RPKLKWDNQKRYGGFLRRQFKVVTRSQEDPNAYSGELLDA

European shrew (*Sorex Araneus*) (XM_004610968)

MEWQVLILILCLSTFPSVSADCPAQCSMCAVQTQNLDKPI

NPLMCFLECQTILTSNTEWEKCKSFLSLFTPFMLGLHGKG

ELGDMSVTSEELYGEQAKPSVELTKTMEKLANILMQENIQ

GRGPSHTYGDLLPKVDEHAVSELMEDSQQYHRALEAGELG

YPSEDTAAAWASTPKDEMKRYGGFLRKYPKRSSEVAGTGD

GQEQEDLHKRYGGFMRRIRPKLKWDNQKRYGGFLRRQFKV

VTRSEEDPNAYSGEVSGL

Tasmanian devil (*Sarcophilus harrisii*) (XM_003757349)

MAALPSMEWHVLALALPLWLGALSSASAQCSAQCSRCSDH

TQGPQDTLDPLICTLECEAAPASGGEWGRCRLILSLLGAL

LPGPRRSGPPAGAPGDPEEEPRTSRGGLGPLGPSRGLPRG

EGLRPPAEAPGVTGGRKAEGKRYGGFLRRYPKRSPPGGAP

HKRYGGFMRRVRPKLRWDNQKRYGASRPRPFKPVTRGEGA

AGALSGEGAA

Platypus (*Ornithorhynchus anatinus*) (XM_029049345)

MEWHLLFLALPLWLGALSSASAQCSSQCSRCSDRTQGLPE

PLDPLICTLECEASPMSGREWGRCRLILSPLGALLGPRSP

SRPEPPAGDPEEEPRARRGGLGLVGPARGPREDEDRRAPA

GAYGAGGGRKAEGKRYGGFLRRYPKSSPPGGDGPHKRYGG

Australian echidna (*Tachyglossus aculeatus*) (XM_038750480)

MERRALALALCLALAAAAAEDCVSRCSACAARVLDYPAPP

PRPPAPGDEEPPGKRYGGFLRTYPKRSGAPGAAGAGQEPA

ELHKRYGGFLRRIRPKLKWDDQKRYGGFLRRQFKVATRAD

EDPSAYSGEVSAL

Emu (*Dromaius novaehollandiae*) XM_026107039

MERQALALALCLALAAAATEDCATRCSTCAARTQDSVESI

RPLMCLLECQGSSPPGAARELCGKALGLLVALADGRELVP

AEDEDEAPPELGPGELAERYGGFAEKLPRGNLFSLLGEDA

HGKGARSKTYSGGKLGERAASGEAQDYPAPEGPGGEEPPG

AAGAGQAPAELHKRYGGFMRRIRPKLKWDNQKRYGGFLRR

QFKVATRADEDPSAYSGEVSD

Okarito brown kiwi (*Apteryx rowi*) (XM_026056408)

MALWSSCMSPHGSVSCSIPWGAPLGADPRGGSCGCRHSSPGPGR

GGGCPLHTNGLWAWISVGSQLWDQIGDALCTLTLPRQRGTMAQR

VLALVLCLSLAATASADCATQCSLCANQARGTESSMQPLMCLWQ

CQGSSPPGAEWESCRKALALLAPLVALAEGTEGTEPSPAEAEDE

AEPEQDPSPEELPLAPAKRYGGFMKKLAKGRLLSLLRDNAHSKG

GLSKKSGGFSHGPGERAAPENYPGPGGGEEPEGAGAEGQELAEL

HKRYGGFMRRIRPKLKWDNQKRYGGFLRRQFKVTTRSDEDPSAY

SGEVLDL

Chicken (*Gallus gallus*) (XM_040650978)

MHEQSLDTENSSGNRAGDNGTSGQVGHAAGGCGSQRWCCH

TLPGSHQGDSAGATAPEHKLLQAVAAGDMARWALELLLCL

SLASVASADCVTQCSLCAARTHGTESSIQPLMCLRECQGS

SPPSPEWETCRKALALLAPLVALAEGTDASPGEAEEDEAE

LEPEQGPGEPPKRYGGFMKKLSKGKLLALLRENAHSKGGL

SKKFGGFGRKLGERAAPQDYPGLGPVGNGDEEPTGAGAAG

QELAELHKRYGGFMRRIRPKLKWDNQKRYGGFLRRQFKVT

TRSDEEPSAYSGEVLDL

Rock pigeon (*Col*u*mba livia*) (M_021282464)

MVLVAPRSRGTAAGASTSPSSLTQGGFWSGTGHCHRHSPS

PSHCIVCSALPGLIHGGSGCGVRWWRSSLALSLALCLSLA

AAASPDCATXMAQTRSTESSVRSLICLQECQGSSPPGPEW

EMCRKALALLAPLVVLAEGTDPSPREAEDEAEPEQELGPG

ELPQAPAKRYGGFMKKMAKGKLLSLLRENAHSKGGLSKKF

GGFGRKPGERAAPEDYPGLGPAGDGGEEPTGAGAEGQELA

ELHKRYGGFMRRIRPKLKWDNQKRYGGFLRRQFKVTMRSD

EDPSAYSGEVSD

Emperor penguin (*Aptenodytes forsteri*) (XM_009282759)

MARRALVLVLCLSLAAVASANCVTQCSLCAVQTHSAESSV

WPLDYPGPAGEGGEKPTGAGAEGQELAELHKRYGGFMRRI

RPKLKWDNQKRYGGFLRRQFKVTTRSDEDPSAYSGEVLDL

Crested ibis (*Nipponia nippon)* (XM_009473421)

MVRWALALVLCLSLAAVASADCVTQCSLCAAQTQGTETSI

RPLMCLWECQGSSPPGPEWDTCRKALALLAPLVALAEGTE

PSPREEEEEEEEAEPEPGPGEQPLVPAKRYGGFMKKMSKG

KLLSLQSAHSKGSLSKKDYPGPAGAGGQEQPMGAEGQELH

KRYGGFMRRIRPKLKWDNQKRYGGFLRRQFKVNTRADEDP

SAYSGEVLDL

Budgerigar (*Melopsittacus undulatus*) XM_034067277

MQPQRWDVHPGPRGSRGAEGRPRRCGRACSGGGGDAAGLI

IHSFPRPDWLCRREQPGPAAGGDSVAIKAGWKAAGGESGG

QPGHHRGTTCQCGLATDDWRSLTPVEGHPSATTRPDLAKR

VLGGTGSSGRQCLWSEMARQALTLALCLSLCAVASADCVT

QCSLCTAQTRGTESSVRPLMCLWECQGFLSPGPQWEMCRK

ALALLAPLVALAEGTDPSPQEAEENEAEPEQDLGPAELPL

APAKRYGGFMKMMSKGKLLSLLRENAHSKGGLSKKFGGFS

RKPGERAAPEDYPGPAGDGDEEPTGAGAEGQELAQLHKRY

GGFLRRIRPKLKWDNQKRYGGFLRRQFKVITRSDEDPSVY

SEEVSDL

Hawaiian crow (*Corvus hawaiiensis*) XM_048322030

MALRVLALGLVLSLAVAASSDCVTQCSLCSAQTDAGVWPL

MCLRECLGSSPPGPEWETCRKALALLAPLVALAEGTDMSP

REVEEEESELQPELGPGELPPVLAKRYGGFMKKMMKGKLL

SLLRENAHSKGGLSKKFGGFGRKPGERAAPEDYPGPAGEG

TKEPTGAGAEGQELAELHKRYGGFMRRIRPKLKWDNQKRY

GGFLRRQFKVAARADEDPSAYSGEVSDV

Common swift (*Apus apus*) XM_051632776

MARRALALALCLSLSAVASADCVTQCSLCAAQSRGAESSV

QPLMCLWECQGSSSPGPEWEMCRKVLALLAPLVALAEGTD

PSPQEAEEDEAQPEQGLGPAELPLAPAKRYGGFMKMMSKA

KLLSLLRENAHSKGGLSKKSGAFSRKPGERAAPEDYPGPA

GDGDEEPTGAGAEGPELAQLHKRYGGFLRRIRPKLKWDNQ

KRYGGFLRRQFKVTTRSDEDPSAYSGEVSDL

Zebra finch (*Taeniopygia guttata)* XM_030288425

**Class Reptilia**

MAWQLLGLSICFCLAPSAFGDCATQCSACALQVQDVEKPI

KPLICSLECQGSLPSRAEWEKCRESLSTLIPFLMEEEEAK

RPFQLEEEEEVPQEPYSSLLAKRYGGFMKKLDKNRIFSLL

RENALNKGGVSKKYGGFFRKVGERAASEPGVEDYPAVLET

GDLAYNGAESDTGSLKDETKRYGGFLRKYPKRGYEMVSTT

EDDGQELEGLHKRYGGFLRRIRPKLKWDNQKRYGGFLRRQ

FKVTTRSEEEPNAYSGEVSD

Green anole (*Anolis carolinensis*) (XM_003226686)

MEWQVLVLALCMGLAHSASADCAAQCSMCAVQTQDLEKPI

SPLICSLECQGSLLFRAEWERCQTALALLTPLMVAVEGQG

LSPLEAEVKAEPEGSPIPGEFPSNLVKRYGGFMKKLDKNK

IFSLLRENAHSKGSMTKKYGGFFRKPGERAASEVAEDYPE

LEARETGDHSEEPEEGNLKDEMKRYGGFLRKYPKRSSALA

PEGEGQELEDLHKRYGGFMRRIRPKLKWDNQKRYGGFLRR

QFKVTTRSDKDPNAYSGEVLD

Geochelone nigra (*Chelonoidis abingdonii)* (XM_032766292)

ALECQGSLLSGAEWEKCQGALSLLTPFLAAAEGQDPSPLE

VEADGSPGLAETSGGPVKRYGGFMKKLDKNKIFSLLRENA

HSKGGLHKKYGGFSRKLGEREALEMPEDDPALEPGDEPED

GGLKDEMKRYGGFLRKYPKRSSEQAAEGDGPKPEDLHKRY

GGFMRRIRPKLKWDNQKRYGGFLRRQFKVTTRSDEDPSIY

SGEVLD

Chinese alligator (*Alligator sinensis*) (XM_006021677)

MQHVGTHAASKRASRASGRVFKTCIWGFGNPSSSGRRPTS

QSARRWKWWNKRGGIGRSRRGSSERTKHRGRRETRRSRRA

AKLPCVPATCSRAGSGLPLLRTRLAEKTAGSVAVWKRRCL

LGRMEWRVLVLAACLGLADPASADCAAQCSACATQTQDAE

QSINPLICALECQDSLLSGAEWEKCQGVLSLFTPFLAAAE

GQDLSPLEAEADGSPGLAETSGGPVKRYGGFMKKLDKNKI

FSLLRENAHSKGGLHKKYGGFSRKLGEREALEMPEDDPAL

EPGDEPEDGGLKDEMKRYGGFLRKYPKRSSEQAAEGDGPK

PEDLHKRYGGFMRRIRPKLKWDNQKRYGGFLRRQFKVATR

SDEDPSIYSGEVLD

American alligator (*Alligator mississippiensis*) (XM_019495253)

MEWQVLVLAACLGLADPASADCAAQCAACAAQTQDAEPSI

NPLICALECQGSLLSGAEWEKCQGALSLLAPFLAAAEGKD

LSPLEAEADGSPGLAEMSGGAVKRYGGFMKKLDKNKIFSL

LRENAHSKGGLHKTYGGFPRKLGEREALEMAEGDPAPEPR

DEPEEGGLKDEMKRYGGFLRKYPKRSSEQAAEGDRPEDLH

KRYGGFMRRIRPKLKWDNQKRYGGFLRRQFKVTTRSDEDP

SIYSEEVLDL

Australian saltwater crocodile (*Crocodylus porosus*) (XM_019548333)

**Class Amphibia**

MQKIMEMGLLVLIMLLSVSAPHATCADCVSWCFSCALQIQ

GTNTDFSPLVCSLQCEGSLTTVTEWQRCENILSPVSAVFE

ITKREQERVPTASDLQEVPVKRYGGFMKKLDKNKFFFNSP

KRESGIYKGEASKPYSGDVLHKYWKKDLLEIPDSEQETEV

NDKLEEQTQARDEKRYGGFLRKYPKRSLEDLQGVQLDRRM

ILDPELGSEQGVEELETEQGVETEHGVADLQKRYGGFMRR

IRPKLKWDNQKRYGGFLRRQFKVTTRSEEDPSTFSGELSN

Common toad (*Bufo bufo*) (XM_040438346.1)

MCTRNRVRLKEIIKTYHDQQRNAYWYQILEVLTGAMERQV

LALILCLGWMPTAWEDCAGQCSNCIEHTKQKEKHINHLVC

TLECEGSLVSSQKWESCRELLSTFLPFLLEPDKRIQDAAG

KQDRDLGMNKPYGDFIRKLEKDTIFSMEENTKGKGNLGQK

SEDSLYSIRSGLVDDPREEKRYGGFLRKYPKRSTEQGPEE

LQKRYGGFMRRIRPKLKWDNQKRYGGFLRRQFRLSTQAEE

PAAFQLKSGTPN

*Geotrypetes seraphini* (XM_033914985)

MEGLVLTLILCLCWMPTAWGDCAVQCSICTEHTKEKEKHI

NQLVCTLECEGFLFSSQKWENCRKLLSTFLPFLLEPDKRI

WDSAGEQDRDLSMIEPGGFIRKLEKDTAFSMEENTRGKGN

LGQKSEDLFHSIRDGIVDGPGEKEKRYGGFLRKYPKRSTE

HGAEELHKRYGGFMRRIRPKLRWDNQKHYGGFLRRQFRLS

TQADEPGAIQLGNGKSK

*Microcaecilia unicolor* (XM_030211619)

**Non-tetrapod Sarcopterygii**

MEWYTLAMILSLGLSYSVKADCSRQCSACSSEGEHTDTHV

DPLVCALECEGSLLSQSEWEKCKEFLKLAPEDLSSSDSNT

VIDPEYNVERQEQQPLSNDQFVSLVKRYGGFLKKIGKNKP

FTRSLTDEDSNTDENMVKRYGGFFRKFGERAAPDITENSQ

EVKSASENEDVTYDMESPNNSPLKEFKRYGGFLRKWNPKR

SADPGEEGIQVQEELQKRYGGFMRRIRPKIRWDNQKRYGG

FLRRHFKITVRSDEDPTPYLDEFLDL

West African lungfish (*Protopterus annectens*) (XM_044089874)

**Class** Actinopterygii

MEWHVLVLVLCLSLSSSIQDDCSSQCLTCAQQTQNEVTQI

NSLVCTLECEGALSPSTELQKCEKVLQMYSAGLFGVNDKA

ETEKEDQPGESPFTNPVKRYGGFLKKLDKSKYYNSSPAQN

SNIKSLLAKKYLYLLRKFDERDIPDMLQDTKVRGEASDNE

EVVYDDATAVNEVKRYGGFLRKFGPKRSLESGEESSEEEL

QKRYGGFMRRVRPKLKWDNQKRYGGFLRRHFKISVRSDEE

PSLYSDEVYYL

Sterlet (*Acipenser ruthenus*) (XM_034056368)

MEWYVLVLILSLPSSTQADCSSHCLQCAQQILNTDIPVNS

LTCTLECEGTLLTTAELDKCGKTQQSRAVGSEFSDEDAGL

RSTPEREDDQDASIANVVKRYGGFIKRIDKNKNKLLTSPW

RENGVYKGAYAFPKKYQDLFRKFGERDLSEFSEDYQGGDV

DSENEMGMFNDDEAAAINKVKRYGGFLRKFGPKTKSKRSD

SREQGSREELQKRYGGFMRRIRPKLNNLNTLKWDNQKRYG

GFLRRHYKLSVRSDEEPSTYDDFG

Chum salmon (*Oncorhynchus keta*) (XM_035739674)

MMEWYVLVLMLSLPSLSQADCSEQCMRCAQQISDLDSAVN

RLTCTLECEGAVPSTSTLDRCEKALQELSDEFAELNPDAD

GERSALNAEDLQEKASNLVKRYGGFIKRIEKNKQKFFASP

WKENAILKGLFAKKYGESLSKLGERDVPSITEDDEGEDVT

AENETGVYDNDVPLNEVKRYGGFLRKFGPKRSNFVENTSP

QVLQKRYGGFMRRIRPKLRWDNQKRYGGFLRRHFKISVRS

DEQPSSYEDNAL

Silver crucian carp (*Carassius gibelio*) (XM_052594757)

MKWSVFVLVLFVTSLHRVRADCANWCSTCNSVLGSSIKPL

TCTLECEGVVLSTNEWESCDKALHSYKLDLFGIVDEALAD

PGTDENEDQSAEALLGKQHDSFVKRYGAFLKKIDRSRINA

KQLAQQNGYKGQNLKHGGLLQKFGERGAAELEENSQELTA

ENLLEKNNDIARTQELKRYGGFMKGFGYKRSAELDDEENQ

DMELQKRYGGFMRRIGRPRYKWDNQKRYGGFLRRHFRVSV

RSDDGDASDYSEEGSDL

Smaller spotted catshark (*Scyliorhinus canicula*) (XM_038803188)

MEWSMFVLALSLGSLYTVHGDCVKWCSACNLVLDSSIKPL

ACTLECEGVVLSTDEWEKCDRALQLYNSEPLGIVDKVLAD

PVTDEKEVKSLEALTGTQHNGFAKRYGGFMKKVDKSRVYA

QALAQENAYQGPNLKYGGLLQKFGEKGCIXLGRGAHRSLQ

LQWENMQADDDDTPSTEELKRYGGFMKGYGNKRSEELADE

ENQNVELQKRYGGFMRRXGRPRYKWDNXKRYGGFLRRNFR

VSLRSDDGEANDYTEEASD

Smalltooth sawfish (*Pristis pectinata*) (XM_052031520)

MMQWNVLVLVFTLTSLHTARADCTSQCSVCSSVLDSSIDPL

ICTLECEGVELSAREWERCEKALHTYKLDILGNDNEAFTIP

NAEEKEGESEEALLGKQYGNLLKPYGSFLRKLDKNRLYAKA

LAQHDTNDKGRVPKKYREYLKKFGERSVPVLEKGSRELTDA

TEDLSEENGDGPEPQERYGGFTKGFGYKRRAEPGDGENQDV

ADLQKRYGGFMRRVGRPRFKWDNQKRYGGFLRRHFRVSVRS

DDDADAYSEEVSDL

Elephant shark (*Callorhinchus milii*) (XM_007911718)

**SUPPLEMENTARY FIGURE 2.** Deduced structures of preprodynorhin

Key:

Green highlight indicates enkephalin motif

Light blue highlight indicates peptide sequences found in neuropeptides along with enkephalin motif

Pink highlight indicates basic amino-acid residues pairs

Yellow highlight indicates degenerate enkephalin motif e.g. lacking two basic amino-acid residue pairs on either N or C sides of the enkephalin motif or lacking enkephalin motif

Blue highlight other amino acid residues
