# Supplementary material for: Avian opioid peptides: evolutionary considerations, functional roles and a challenge to address critical questions
Source: Front Physiol. 2023 Jun 6;14:1164031. doi: 10.3389/fphys.2023.1164031 (PMC10280075; doi:10.3389/fphys.2023.1164031)
Supplement: Supplementary file 6 [file DataSheet1.DOCX]

**Class Mammalia**

MARFLTLCTWLLLLGPGLLATVRAECSQDCATCSYRLVRPADINFLACVMECEGKLPSLKIWETCKELLQLSKPELPQDGTSTLRENSKPEESHLLAKRYGGFMKRYGGFMKKMDELYPMEPEEEANGSEILAKRYGGFMKKDAEEDDSLANSSDLLKELLETGDNRERSHHQDGSDNEEEVSKRYGGFMRGLKRSPQLEDEAKELQKRYGGFMRRVGRPEWWMDYQKRYGGFLKRFAEALPSDEEGESYSKEVPEMEKRYGGFMRF

Human (*Homo sapiens*) (BC032505)

MARFLTLCTWLLLLGPGILATVRAECSQDCATCSYRLVRPAD

INFLACVMECEGKLPSLKIWETCKELLQLSKPELPQDGTSTL

ESSK-PEESHLLAKRYGGFMKRYGGFMKKMDELYPMEPEEEA

NGSEILAKRYGGFMKKDAEEDDSLANSSDLLKELLETGDNRE

SHHQDGSDNEEEVSKRYGGFMRGLKRSPQLEDEAKELQKRYG

GFMRRVGRPEWWMDYQKRYGGFLKRFAEA-LPSDEEGESYSK

EVPEMEKRYGGFMRF

Bonobo (*Pan paniscus*) (XM_003823230)

MARFLTLCTWLVALGPGLLATVTADCGQDCAAC-ALLARPAD

LSAETCTLECEGKLSSLNTWGICNELLHPSKVEL--PRDSSR

GSKWDDVDWQSYMKRYGGFMKRYGGFMKRYGGFMKKMDELYEAEPEE--

NQGETLAKRYGGFMKKDAEEDTLANSSDLLRELLGTGERGEG

IH-QYNRED--EVSKRYGGFMKSAKRMPEMEDEVKELQKRYG

GFMRRVGRPEWWMDYQKRYGGFLKRFAEP-LPSEEEGESYSK

WYPKVDKRYGGFMKP

European shrew (*Sorex Araneus*) (XM_012936042)

MALFLRLCSLLLALSPGLFLTIRAECSKDCTSCTYRLGQHAD

INPLACTLECEGKLPSAKAWDTCKELLQLTKMDLSQEGTNNQE

ES-K-QDDSHLLAKKYGGFMKRYGGFMKKMDELYRVEPEDEM

NGGEVLAKRYGGFMKKDSDDDALANSSDLLLKELLGTGDNSEA

GRYQAGNENEEEVSKRYGGFMRSYKRSPELEDEAKELQKRYG

GFMRRVGRPEWWLDYQKRYGGFLKRFADS-LPSDEEGESYSK

EVPEMEKRYGGFMR

Gray short-tailed opossum (*Monodelphis domestica*) (XM_001368487)

MALFLKLCSLLLALSPGLFLTIRAECSKDCTSCTFRLGQHAD

INPLACTLECEGKLPSAKAWDTCKELLQLTKMDLSQESTNNQG

GDSDKPQEASHLLAKKYGGFMKRYGGFMKKMDELYHMEPEDET

GHYREGNENEEEMSKRYGGFMRSYKRSPELED----------

NGGEILAKRYGGFMKKDSDDDALANSSDLLLKELLGTGDISEA

EAKELQKRYGGFMRRVGR

Tasmanian devil (*Sarcophilus harrisii*) (XM_012541312)

MALVLRLCGLLLAVGPGLVLAGRAGCGKDCAACAHRLGRRAD

INPLACTLECEGKLPSAKAWETCKDLLQLTKLDLSQEGSNVL

QQEGSDILASHLLAKKYGGFMKRYGGFMKKTDELYRTEPEDET

AGDILAKRYGGFMKKDSDDEALANSSDLLKELLGGGDDPET

AHYRGVNENDGDVSKRYGGFMRSVKRSPELEDEAKELQKRYG

GFMRRVGRPEWWLDYQKRYGGFLKRFADS-LPSEEDEESYSK

EVPEMDKRYGGFMRF

Platypus (*Ornithorhynchus anatinus*) (XM_007667187)

--PLACTLECEGKLPSAKAWETCKDLLQLTKLDLS-------

GDKKELDENHLLAKKYGGFMKRYGGFMKKMDELYHPESEDEA

PAGDVLAKRYGGFMKKDSDDGALADSSDLLKELLGGGDSPQT

GHYQEGGDNDEEVGKRYGGFMRGYKRSPELEDEAKALQKRYG

GFMRRVGRPEWWLDYQKRYGGFLKRFADS-LPSEEDEESYSK

EVPEMDKRYGGFMRF

Australian echidna (*Tachyglossus aculeatus*) (XM_038766685)

**Class Aves**

MWNLLLNTVPDKHSQDPRGKGEKVKKRPPENLAGSRKKSGSRAASVAVPPSAL

LAFTACTLECEGKLPSAKAWETCKELLQLTKLDLSEDGNIAP

GDKKELDENHLLAKKYGGFMKRYGGFMKKMDELYRVEPEEEA

NGGEILAKRYGGFMKKDSDDDALANSSDLLNELLGTGDNPEA

GHYREINENDGDVSKRYGGFMRSVKRSPELEDEAKELQKRYG

GFMRRVGRPEWWLDYQKRYGGFLKRFADSILPSEEDGESYSK

EIPEIEKRYGGFMRF

Ostrich (*Struthio camelus*) (XM_009673565)

ILPLACTLECEGKLPPAKAWETCKELLQLTKLDLSEDGNSAP

MARLLPLGCALLALSTCLLLRARADCGRQCAACAYRLGPRAR

GDKKELDENHLLAKKYGGFMKRYGGFMKKMDELYGVEPEEEA

DGGEILAKRYGGFMKKDSDDDTLANSSDLLKELLGTGDNPEA

GHYGEINENDGDVSKRYGGFMRSVKRSPELEDEAKELQKRYG

GFMRRVGRPEWWLDYQKRYGGFLKRFADSILPSEEDGESYSK

EIPGMEKRYGGFMRF

Chilean tinamou (*Nothoprocta perdicaria*) (XM_026050114)

MARLLRLGCSLLALSTCLLLRARADCGRDCAACAYRRGPRAD

IHPLACTLECEGKLPSAKAWETCKELLQLTKLDLSEDGNIAP

GDKKELDENHLLAKKYGGFLKRYGGFMKKMDELYRVEPEEEA

NGGEILAKRYGGFMKKDSDDDALANSSDLLKELLGTGDNPEA

GRYREINENDGDVSKRYGGFMRSVKRSPELEDEAKELQKRYG

GFMRRVGRPEWWLDYQKRYGGFLKRFSDSILPSEEDGESYSK

EIPEMEKRYGGFMRF

Okarito brown kiwi (*Apteryx rowi)* (XM_026092298)

MARLLALGCSLLALSTCLLPRARADCGRDCAACAYRLGPRAD

IHPLACTLECEEKLPSAKAWETCKELLQLTKLDLSEDSNIAP

GDKKELDENHLLAKKYGGFMKRYGGFMKKMDELYRVEPEGEA

NGGEILAKRYGGFMKKDSDDDVLANSSDLLKELLGXGDNPEA

GRYREINENDGDVSKRYGGFMRSIKRSPELEDEAKELQKRYG

GFMRRVGRPEWWLDYQKRYGGFLKRFADSILPSEEDGESYSK

EIPEMEKRYGGFMRF

Emu (*Dromaius novaehollandiae*) (XM_026115758)

MALLLRLGCSLLALSTCLLPRARADCGRDCAACAYRLGPRAD

IHPLACTLECEGKLPSAKAWETCKELLQLTKLDLPEDGNAAP

GDKKELDENHLLAKKYGGFMKRYGGFMKKMDELYHPESEDEA

NGGEILAKRYGGFMKKDSDDDALANSSDLLKELLGTGDNPEA

AHYRGVNENDGDVSKRYGGFMRSVKRSPELEDEAKELQKRYG

GFMRRVGRPEWWLDYQKRYGGFLKRFADSILPSEEDGETYSK

EVPEMEKRYGGFMRF

Chicken (*Gallus gallus*) (XM_040664746)

MALLLRLGCSLLALSTCLLPRVRADCGRECATCAYRLGPRAD

-------------------------MLQLTKLDLSEDGNAAP

GDKKELDENHLLTKKYGGFMKRYGGFMKKMDELYHPESEDEA

NGEEILAKRYGGFMKKDSDDDALANSSDLLKELLGAGDNPEA

AHYRGINENDGDVSKRYGGFMRSIKRSPELEDEAKELQKRYG

GFMRRVGRPEWWLDYQKRYGGFLKRFADSILPSEEDGETYSK

EVPEMEKRYGGFMRF

Turkey (*Meleagris gallopavo*) (XM_00320506)0

MRDWLEPSSCSPPCQALGTHPTSAKMQGEDQDETKGSNDRG-

IHPLACTLECEGKLPSAKAWETCKELLQLTKLDLSEDSSIAP

GDKKELDENHLLAKKYGGFMKRYGGFMKKMDELYRVEPEDEA

NGGEILAKRYGGFMKKDSDDDALANSSDLLKELLGAGDNPEA

GRYREISENDGDINKRYGGFMRSVKRSPELEDEAKELQKRYG

GFMRRVGRPEWWLDYQKRYGGFLKRFADSILPSEEDGETYSK

EVPEMEKRYGGFMRF

Mallard (*Anas platyrhynchos*) XM_027452260

MPRPLQKDMKAFRAALPEELSFQATRREARSWPFKSKKSYDKVTVRCLLLSSHSQQTP

SVFQACTLECEGKLPSAKAWETCKELLQLTKLDLSEDGSVAP

GDKKELDENHLLAKKYGGFMKRYGGFMKKMDELYRVEPEDEA

NGGEILAKRYGGFMKKDSDDDALANSSDLLKELLGTGDNPEA

GHYREISENDGDINKRYGGFMRSIKRSPELEDEAKELQKRYG

GFMRRVGRPEWWLDYQKRYGGFLKRFADSILPSEEDGETYSK

EVPEMEKRYGGFMRF

Swan goose (*Anser cygnoides*) (XM_013185865)

YGEADEPKSAEYLQHFQQPWLDFFCPVLAVHSCAYRLGPRAS

IHPLACTLECEGKLPSAKAWETCKDLLQLAKLDLSEDGNIAP

GDKKELDENHLLAKKYGGFMKRYGGFMKKMDELYRVEPEDEA

NGGEILAKRYGGFMKKDSDEDALANSSDLLKELL--GDNPEV

GHYQEINENDGDVSKRYGGFMRSIKRSPELEDEAKELQKRYG

GFMRRVGRPEWWLDYQKRYGGFLKRFADSIRPSEEDGETYSK

DVPEMEKRYGGFMRF

Rock pigeon (*Columba livia*) (XM_013368343)

MALLLRLGCSLLALSTCLLPRVRGDCGRDCAGCAYRLGPRAG

IHPLACTLECEGKLPSAKAWETCKELLQLAKLDLSEDGNIAP

GDKKELDENHLLAKKYGGFMKRYGGFMKKMDELYRVEPEDEA

NGGEILAKRYGGFMKKDSDDDALANSSDLMKELLGTGDNPEA

GHYRDINENDGDVSKRYGGFMRSIKRSPELENEAKELQKRYG

GFMRRVGRPEWWLDYQKRYGGFLKRFADSILPSEEDGETYSK

EVPEMEKRYGGFMRF

Anna's hummingbird (*Calypte anna*) (XM_008499689)

MCSFGNRGLLKLIEKGENLFLLXFKAISKSDXSQDPILYSITITHSNNR

IHPWACTLECEGKLPTAKAWETCKELLQLAKLDLSEDANIAP

GEKKELDENHLLAKKYGGFMKRYGGFMKKMDELYRVEPEDEA

NGGEMLAKRYGGFMKKDSDDDALANSSDLLKELLGAGDSPEA

GHYREINENDGDVSKRYGGFMRSIKRSPELEDEAKELQKRYG

GFMRRVGRPEWWLDYQKRYGGFLKRFADSILPSEEDGETYSK

EVPEMEKRYGGFMRF

Macqueen's bustard (*Chlamydotis macqueenii*) (XM_010130600)

----ACTLECEGKLPSAKAWETCKELLQLAKLDLSEDGNIAP

GDKKELDENHLLAKKYGGFMKRYGGFMKKMDELYRVEPEDEA

NGGEILTKRYGGFMKKDSDDDALANSSDLLKELLGTGDNPEA

GHYREINENDGDVSKRYGGFMRSIKRSPELEDEAKELQKRYG

GFMRRVGRPEWWLDYQKRYGGFLKRFADSILPSEEDGETYSK

EVPEMEKRYGGFMRF

Speckled mousebird (*Colius striatus*) (XM_010195765)

MALLLRLGCSLLALSSCLLLGARADCGRDCAACAFRPGPRAG

----ACTLECEGKLPSAKAWETCKELLQLVKLDLSEDGSIAP

GDKKELDENHLLAKKYGGFMKRYGGFMKKMDELYRVEPEDEA

NGGEILAKRYGGFMKKDSDDDALANSSDLLKELLGTGDNPEA

GHYREINENDGDVSKRYGGFMRSVKRSPELEDEAKELQKRYG

GFMRRVGRPEWWLDYQKRYGGFLKRFADSILPSEEDGETYSK

EVPEMEKRYGGFMRF

Common cuckoo (*Cuculus canorus*) (XM_009563289)

----ACTLECEGKLPSAKAWDTCKELLQLAKLDFPEDGNIGP

GDKKELDENHLLAKKYGGFMKRYGGFMKKMDELYRVEPEDEA

NGGEILAKRYGGFMKKDSDDDTLANSSDLLKELLGTGDNPEA

GHYREINENDGDVSKRYGGFMRSVKRSPESEDEAKELQKRYG

GFMRRVGRPEWWLDYQKRYGGFLKRFADSILPSEEDGETYSK

EVPEMEKRYGGFMRF

Bar-tailed trogon (*Apaloderma vittatum*) (XM_009865217)

----ACTLECEGKLPSAKAWETCKELLQLAKLDLAEDGNIAP

GDKKELDENHLLAKKYGGFMKRYGGFMKKMDELYRVEPEDEA

NGGEILAKRYGGFMKKDSDDDALANSSDLLKELLGTGDNPEA

GHYREINENDGDVSKRYGGFMRSIKRSPELEDEAKELQKRYG

GFMRRVGRPEWWLDYQKRYGGFLKRFADSILPSEEDGEAYSK

EVPEMEKRYGGFMRF

Red-crested turaco (*Tauraco erythrolophus*) (XM_009988038)

---- ACTLECEGKLPSAKAWETCKELLQLAKLDLSEDGNVAP

GDRKEPDENHLLAKKYGGFMKRYGGFMKKMDELYRVEPEDEA

NGGEILAKRYGGFMKKDSDDDALANSSDLLKELLGTGDNPEA

GHYREGNENDGDISKRYGGFMRSVKRSPELEEEAKELQKRYG

GFMRRVGRPEWWLDYQKRYGGFLKRFADSILPSEEDGETYSK

EVPEMEKRYGGFMRF

Rhinoceros hornbill (*Buceros rhinoceros*) (XM_010144053)

MGLNRQ------------------------------------

----ACTLECEGKLPSAKAWETCKELLQLAKLDLSEDGNVAP

GDKQELDENHLLAKKYGGFMKRYGGFMKKMDELYRVEPEDEA

NGGEILAKRYGGFMKKDSDDNALANSSDLLKELLGTEDNPEA

GHYREINENTGDVSKRYGGFMRSVKRSPELEDEAKELQKRYG

GFMRRVGRPEWWLDYQKRYGGFLKRFADSILPSEEDGETYSK

EVPEMEKRYGGFMR

Red-throated loon (*Gavia stellata*) (XM_009820800)

MGLNRQ------------------------------------

----ACTLECEGKLPSAKAWETCKELLQLAKLDLSEDGNIAP

GDKKELDENHLLAKKYGGFMKRYGGFMKKMDELYRVEPEDEA

NRGEILAKRYGGFMKKDSDDDTLANSSDLLKELLGTGNNPEA

GHYREINENDGDVSKRYGGFMRSVKRSPELEDEAKELQKRYG

GFMRRVGRPEWWLDYQKRYGGFLKRFADSILPSEEDGETYSK

EVPEMEKRYGGFMRF

Killdeer (*Charadrius vociferus*) (XM_009892789)

----ACTLECEGKLPSAKAWETCKELLQLAKLDLSEDGNTAP

GDKKELDENHLLAKKYGGFMKRYGGFMKKMDELYRVEPEGEA

NGGEILAKRYGGFMKKDSDDDALANSSDLLKELLGTGDNPEA

EHYREINENDGDVSKRYGGFMRSIKRSPELEDEAKELQKRYG

GFMRRVGRPEWWLDYQKRYGGFLKRFTDSILPSEEDGETYSK

EVPEMEKRYGGFMRF

Dalmatian pelican (*Pelecanus crispus*) (XM_009486988)

MGLNRQ------------------------------------

----ACTLECEGKLPSAKAWETCKELLQLAKLDLSEDGNIAP

GDRKDLDENHLLAKKYGGFMKRYGGFMKKMDELYRVEPEDEA

NGGEILAKRYGGFMKKDSDDDALANSSDLLKELLGTGDNPEA

GHYREINENDGDVSKRYGGFMRSIKRSPELEDEAKELQKRYG

GFMRRVGRPEWWLDYQKRYGGFLKRFADSILPSEEDGEIYSK

EVPEMEKRYGGFMR

Emperor penguin (*Aptenodytes forsteri*) (XM_009289362)

MALFLRLGCSLLALSTCLLPRARADCGRDCAACAYRLGPRAG

IHPLACTLECEGKLPSAKAWETCKELLQLAKLDLSEDGSITP

GDKKELDENHLLAKKYGGFMKRYGGFMKKMDELYRVEPEDEG

NGGEILAKRYGGFMKKDSDDDALANSSDLLKELLGTGDNPEA

GQYRELSEKEGDVSKRYGGFMRSVKRSPEMEDEAKELQKRYG

GFMRRVGRPEWWLDYQKRYGGFLKRFADSILPSDDDGETYSK

EGPEMEKRYGGFMRF

Downy woodpecker (*Dryobates pubescens*) (XM_009904107)

MALLLRLGCSLLALSTCLLLGVRADCGRDCAGCAYRLGPRAG

IHPLACTLECEGKLPSAKAWETCKELLQLVKLDLSENGNVAP

GDKKELDENHLLAKKYGGFMKRYGGFMKKMDELYRVEPEDEG

NGGEILAKRYGGFMKKDSDDDALDNSSDLLKELLGTGDNPEA

GHYREINENDGDVSKRYGGFMRSIKRSPGLEDEAKELQKRYG

GFMRRVGRPEWWLDYQKRYGGFLKRFADSILPSEEDGEAYSK

EVPEMEKRYGGFMRF

Barn owl (*Tyto alba*) (XM_042786250)

MAMLLRLGCSLLALSTCLLPRARADCGRDCTACAYRLGPRAG

IHPLACTLECEGKLPSAKAWETCKELLQLAKLDLSEDGNISP

GEKKELDENHFLAKKYGGFMKRYGGFMKKMDELYQVEPEDEA

NGGEILAKRYGGFMKKDSDDDALANSSDLLKELLGTGDNPEA

GHYREISENDGDVSKRYGGFMRSVKRXPELEDEAKELQKRYG

GFMRRVGRPEWWLDYQKRYGGFLKRFADSILPSEEDGETYSK

EVPEMEKRYGGFMRF

California condor (*Gymnogyps californianus*) (XM_050892863).

MALLLRLGCSLLALSTCLLPRVRADCGRDCAACAYRLGPRAG

IHPLACTLECEGKLPSAKAWETCKELLQLAKLDLSEDGNIAP

GDKKELDENHLLAKKYGGFMKRYGGFMKKMDELYRVEPEDEA

NGGEILAKRYGGFMKKDSDDDALANSSDLLKELLGTGDNPEA

GHYREINENDGDVSKRYGGFMRSIKRSPELEGEAKELQKRYG

GFMRRVGRPEWWLDYQKRYGGFLKRFADSILPSEEDGETYSK

EVPEMEKRYGGFMRF 24.

Golden eagle (*Aquila chrysaetos*) XM_030012713)

MALLLRLGCSLLALSTCLLPRARADCGRDCAACAYRLGPRAG

IHPLACTLECEGKLPSAKAWETCKELLQLTKLDLSEDGNISP

GDKKELDENHLLAKKYGGFMKRYGGFMKKMDELYRAEPEDEA

NGGEILAKRYGGFMKKDSDDDALANSSDLLKELLGTGDNPEA

AHYREINENDGDVSKRYGGFMRSIKRSPELEDEAKELQKRYG

bGFMRRVGRPEWWLDYQKRYGGFLKRFADSILPSEEDGETYSK

EVPEMEKRYGGFMRF

Hawaiian crow (*Corvus hawaiiensis*) (XM_048287048)

MALLLRLGCSLLALSTCLLPRARADCGRDCAACAYRLGPRSG

IHPLACTLECEGKLPSAKAWETCKELLQLAKLDLSEDGNIAP

GDKKELDENHLLAKKYGGFMKRYGGFMKKMDELYRAEPEDEA

NGGEILAKRYGGFMKKDSDDDALANSSDLLKELLGTEDNPEA

AHYRDINE-DGDVSKRYGGFMRSIKRSPELEDEAKELQKRYG

GFMRRVGRPEWWLDYQKRYGGFLKRFADSILPSEEDRETYSK

EVPEMEKRYGGFMRF

Wire-tailed manakin (*Pipra filicauda*) (XM_027749057)

MALLLRLGCSLLALSACLLPRARADCGRDCAACAYRLGPRAG

IHPLACTLECEGKLPSAKAWETCKELLQLAKLDLSEDGNIAP

GDKKEVDENHLLAKKYGGFMKRYGGFMKKMDELYRAEPEDEA

NGGEILAKRYGGFMKKDSDDDALANSSDLLKELLGTGDSPEA

AHYREINENDGDVSKRYGGFMRSIKRSPELEDEAKELQKRYG

GFMRRVGRPEWWLDYQKRYGGFLKRFADSILPSEEDGETYSK

EVPEMEKRYGGFMR

Bengalese finch (*Lonchura striata*) (XM_021555675)

MALLLRLGCSLLALSTCLLPSARADCGRDCAACAYHLGPRAG

IHPLACTLECEGKLPSAKAWETCKELLQLAKVDLSEDGNIAP

GDKKELDENHLLAKKYGGFMKRYGGFMKKMDELYRAEPEDEA

NGGEILAKRYGGFMKKDSDDDALANSSDLLKELLGTGDNPEA

AHYREVNENDGDVSKRYGGFMRSTKRSPELEEEAKELQKRYG

GFMRRVGRPEWWLDYQKRYGGFLKRFADSILPSEEDGETYSK

EVPEMEKRYGGFMRF

Common canary (*Serinus canaria*) (XM_030239219)

MALLLRLGCSLLALSTCLLPRARADCGRDCAACAYRLGARAG

IHPLACTLECEGKLPSAKAWETCKEFLQLVKLDFSEDGNIPP

GDKKELDENHLLAKKYGGFMKRYGGFMKKMDELYRAEPEDEA

NGGEILAKRYGGFMKKDSDDDALANSSDLLKELLGTGDNPEA

AHYREISENDGDVSKRYGGFMRSVKRSPELEDEAKELQKRYG

GFMRRVGRPEWWLDYQKRYGGFLKRFADSILPSEEDGETYSK

EVPEMEKRYGGFMRF

Barn swallow (*Hirundo rustica*) (XM_040064844)

**Class Reptilia**

MASLLRRCCWLLALSTCLALTVRAACSRDCASCASRLGHH

AEINPLACTLECEGKLPSSKAWETCKEILQLTKLDPSAEG

NSAPVDNKKEQDENHLLAKKYGGFMKRYGGFMKKMDELYH

VEPEEETTGGEILAKRYGGFMKKDSDDDTLANSSDLLKEL

GHYREINENDGEISKRYGGFMRSIKRSPELEDEAKELQKR

YGGFMRRVGRPEWWLDYQKRYGGFLKRFTDSVLPSEEDGE

SYSKEVPEMEKRYGGFMRF

American alligator (*Alligator mississippiensis*) (XM_006270885)

MASLLRRCCWLLALSTCLALTVRAACSRDCASCAYRLGHH

INPLACTLECEGKLPSSKAWETCKEILQLTKLDLSAEGNS

VDNKKEQDENHFLAKKYGGFMKRYGGFMKKMDELYHVEPE

EETAPAETGGEILAKRYGGFMKKDSDDDTLANSSDLLKEL

LGTGDNNEAGHYREISENDGEVSKRYGGFMRSIKRSPELE

DEAKELQKRYGGFMRRVGRPEWWLDYQKRYGGFLKRFTDS

VLPSEEDGESYSKEVPEMEKRYGGFMRF

Australian saltwater crocodile (*Crocodylus porosus*) (XM_019548337)

MTLLLRCHFLLLAFSTYFILTVQAECSKDCASCIQRLAYH

ANINPLACTLECEGKLPSAKAWETCKELLQLAKLDLSQEG

NGAIGDNTKQDESHLLAKKYGGFMKRYGGFMKKMDELYDI

EPEEDFSRAGIVAKRYGGFMKKDSDDDTLANSSDLLKELL

ETGENAEPGRYRGLNDNDGEIIKRYGGFMRSIKRSPESED

DAKELQKRYGGFMRRVGRPEWWLDYQKRYGGFLKRFADSI

LPSEEDGEGYSKEVPEMEKRYGGFMRF

Green anole (*Anolis carolinensis)* (XM_003228481)

MYPMALLLRLCGLFLALSSRLVWTVQADCSKDCASCSYRL

GHHAEINPLACTLECEGKLPSAKAWETCKELMQLTKLDLS

EEGNSAPGDNKKEQDENHLLAKKYGGFMKRYGGFMKKMDE

LYHVEPEEETNGGEILTKRYGGFMKKDSNDDALANSSDLL

KELLGTGANAEASHYRQINDNDGEVSKRYGGFMRSFKRSP

ELEDEAKELQKRYGGFMRRVGRPEWWLDYQKRYGGFLKRF

TDSFLPSDEDGESYSKEVPEMEKRYGGFMR

Green sea turtle (*Chelonia mydas*) (XM_007054508)

**Class Amphibia**

MQSMALVTRYCCLVLAVTTYLAVAVQADCSKDCTSCAYHM

GQATEVNSLACTLECEGKLPTAKAWGTCKELLQTAKDNSQ

EADKEQGIDTHLLAKKYGGFMKRYGGFMKKKMDELYHVEP

EEESNGGEILAKKYGGFMKKEYDSDTSDLLRELLGTVGDP

ESGLYRDNNSETPGEVNKRYGGFMRDYRRSSDNEDEGREL

QKRYGGFMRRVGRPEWWQDYQKRYGGFMRRFADSLLPSDE

DGESYSKEIPEMDKRYGGFMRF

Common toad (*Bufo bufo*) (XM_040432544)

MALLLRHCSFLVALNACLLVTVRTECNKDCASCTYQLGHH

AEINPVACTLECEGKLPSSKAWDSCKELLQLAKLDLSQER

EKDHENDENLILAKKYGGFMKRYGGFMKKMDERYHIEPEE

ENNDGEILPKSYGGFLKKMDELYHMEPEEENNDGEILPKR

YGGFMKKGSDRDVSDLLKELLGTDGGDNSETGHYRDSYHR

LGEVSKRYGGFMRGFKRSPELEDEAKELQKRYGGFMRRVG

RPEWWLDYQKRYGGFMKRFPDSFLPSDEDAESYSKEIPEM

EKRYGGFMRF

Microcaecilia unicolor (XM_030190154)

MALLVKCSCLLLVLCACLILAVWAECSKDCAHCTYHLGQQ

AEINPLSCTLECEGKLSSTKTWDMCKELLQAGKSEGTQEG

ESTSTENEKESLERLLAKRYGGFMKRYGGFMKKMDELYHL

EPESENNGREILAKRYGGFMKKDPETGSLTDSSDLLRDLL

FGGDNREGDYYMENTGKENNVMKRYGGFMRSLKRSTDQED

MAKDLQKRYGGFMRRVGRPEWKLDNQKRYGGFMRRFTDPL

FSEEDSEMNSTEDPDTEKRYGGFMGY

West African lungfish (*Protopterus annectens*) (XM_044065545)

MALTEKCTGWMLLLSACFILTVQAECGKDCAYCIYHLQQG

RHAEINTLTCTLECERKLPTTKTWEVCKGILQGNRAEMST

EQENDPLEGVNQENDDHQLAKRYGGFMKRYGGFTKKMAEL

YNVDPDDDNDGSEILAKRYGGFMKKDVESGSQADTAGLLR

EILNAGDSEVDSNSDHDGEITKRYGGFMRSIKRSLDLEDG

IKELQKRYGGFMRRVGRPDWKQNIKRYNGFSKQPENKDSK

EITSEEVEKRYGGFMGY

Sterlet (*Acipenser ruthenus*) (XM_033993708) YGGFM YGGFT YGGFMRSI YGGFMGY

MAVPGNSLWRLLLCAYFALTVGADCEKDCALCLNRILGQQ

TAINTLTCSIECEGSLDTTKLRLCRDVLLEEEPVAVDEIK

QEEVEGEQHQLAKKYGGFMKRYGGFMIRRSPXPVQDGGVQ

GGQNSPVAEEEDIRLEILKILNSEAEAQRDGELAKRYGGF

MRRGGDFGALDVAGRPLKKRYGGFMRRVGRPEWLEDQKNK

GGLLKRSWEGQGVDSPLSEIQKKYGGFMD

Chum salmon (*Oncorhynchus keta)* (XM_035799280)

MALTVNWWTLALSACLVLMVRAECSRDCALCVYRLFRQRT

EMDTLTCSLQCEGTVDSRKLEICKKILSEEDSLALDTLRQ

QEESADHLLTKKYGGFMKRYGGFMIKKAAEIGMGAPPEND

GIEVISKKYGGFIKKDKVEGGVEDQQVELLREILRVGLTS

ESDEHHDRDMVKRYGGFMRSVQGLGEQGRDLHKRYGGFMR

RVGRPDWLDNQKSSGFLKHTWEDGGETVLPNMQKRYGGFM

D

Silver crucian carp (*Carassius gibelio*) (XM_052547787)

MALLWKCHCLVAFLCASFLGVGADCDQDCAYCAYHLAGHL

TEFNPLSCTLECEGKLPSGKAWGMCKELEEVNKPHDDSES

SPESDKEKEAQRLLLNKRYGGFMKRYGGFMKKADSGDTYI

SEVDDENKGREILSKRYGGFMKKDIESPSSVDSADILREL

LNLNELNERKHYLDHSDSDNRSEIMKRYGGFMNGFKRSPE

IEDLPELQKRYGGFMRRFGKPEYQKRYGGFMKRWNDALIP

SDEDGEIYSKEVPELEKRYGGFMRI

Smaller spotted catshark (*Scyliorhinus canicula*) (XM_038809401)

MALLWKCHYLVAVFCASFAGVGADCDQECANCAYHLAGHS

TEFNSLSCMLECEGKMPSAKAWGICKELAEVNKPHDDSQS

AAENDKEKEEQRQLLTKRYGGFMKRYGGFMKKGDTSDTYI

SDADDENKGREILNKRYGGFMKKDTESASSVDTTDVLREL

LNLSELNDRKHYLDQSDSDSRSEIMKRYGGFMNGFKRNQE

FEDLPELQKRYGGFMRRFGKPDYQKRYGGFMKRWNDALVP

SDEDGEIYSKEVPELEKRYGGFMR

Thorny skate (*Amblyraja radiata*) (XM_033019756)

**SUPPLEMENTARY FIGURE 1.** Deduced structures of preproenkephalin

Key:

Green highlight indicates enkephalin motif

Light blue highlight indicates peptide sequences found in neuropeptides along with enkephalin motif

Pink highlight indicates basic amino-acid residues pairs

Yellow highlight indicates degenerate enkephalin motif e.g. lacking two basic amino-acid residue pairs on either N or C sides of the enkephalin motif or lacking enkephalin motif

Blue highlight other amino acid residues
